# An annotated list of fish parasites (Isopoda, Copepoda, Monogenea, Digenea, Cestoda, Nematoda) collected from Snappers and Bream (Lutjanidae, Nemipteridae, Caesionidae) in New Caledonia confirms high parasite biodiversity on coral reef fish

**DOI:** 10.1186/2046-9063-8-22

**Published:** 2012-09-04

**Authors:** Jean-Lou Justine, Ian Beveridge, Geoffrey A Boxshall, Rodney A Bray, Terrence L Miller, František Moravec, Jean-Paul Trilles, Ian D Whittington

**Affiliations:** 1 UMR 7138 Systématique, Adaptation, Évolution, Muséum National d’Histoire Naturelle, Case postale 51, 55, rue Buffon, 75231 Paris cedex 05, France; 2 Department of Veterinary Science, University of Melbourne, Veterinary Clinical Centre, Werribee, 3030, Victoria, Australia; 3 Department of Zoology, Natural History Museum, Cromwell Road, London, SW7 5BD, UK; 4 Biodiversity Program, Queensland Museum, PO Box 3300, South Brisbane, Queensland, 4101, Australia; 5 Institute of Parasitology, Biology Centre, Academy of Sciences of the Czech Republic, Branišovská, 31 370 05, České Budějovice, Czech Republic; 6 Équipe Adaptation écophysiologique et Ontogenèse, UMR 5119 (CNRS-UM2-IRD-UM1-IFREMER), Université Montpellier 2, Place Eugène Bataillon, 34095, Montpellier cedex 05, France; 7 Monogenean Research Laboratory, The South Australian Museum, Adelaide 5000, & Marine Parasitology Laboratory, & Australian Centre for Evolutionary Biology and Biodiversity, The University of Adelaide, North Terrace, Adelaide, 5005, South Australia, Australia

**Keywords:** Biodiversity, Coral reefs, Parasites, Coextinction, Lutjanidae, New Caledonia, South Pacific

## Abstract

**Background:**

Coral reefs are areas of maximum biodiversity, but the parasites of coral reef fishes, and especially their species richness, are not well known. Over an 8-year period, parasites were collected from 24 species of Lutjanidae, Nemipteridae and Caesionidae off New Caledonia, South Pacific.

**Results:**

Host-parasite and parasite-host lists are provided, with a total of 207 host-parasite combinations and 58 parasite species identified at the species level, with 27 new host records. Results are presented for isopods, copepods, monogeneans, digeneans, cestodes and nematodes. When results are restricted to well-sampled reef fish species (sample size > 30), the number of host-parasite combinations is 20–25 per fish species, and the number of parasites identified at the species level is 9–13 per fish species. Lutjanids include reef-associated fish and deeper sea fish from the outer slopes of the coral reef: fish from both milieus were compared. Surprisingly, parasite biodiversity was higher in deeper sea fish than in reef fish (host-parasite combinations: 12.50 vs 10.13, number of species per fish 3.75 vs 3.00); however, we identified four biases which diminish the validity of this comparison. Finally, these results and previously published results allow us to propose a generalization of parasite biodiversity for four major families of reef-associated fishes (Lutjanidae, Nemipteridae, Serranidae and Lethrinidae): well-sampled fish have a mean of 20 host-parasite combinations per fish species, and the number of parasites identified at the species level is 10 per fish species.

**Conclusions:**

Since all precautions have been taken to minimize taxon numbers, it is safe to affirm than the number of fish parasites is at least ten times the number of fish species in coral reefs, for species of similar size or larger than the species in the four families studied; this is a major improvement to our estimate of biodiversity in coral reefs. Our results suggest that extinction of a coral reef fish species would eventually result in the coextinction of at least ten species of parasites.

## Background

Parasites probably constitute the least known component of biodiversity in coral reefs, which are considered some of the most diverse ecosystems on the planet [1]. An early evaluation of parasite biodiversity of fish of the Great Barrier Reef (GBR) in Australia proposed a number of 20,000 parasites (all groups included) in the 1,000 fish species believed to exist in the area at this time; however, this evaluation, published as short papers [2,3] was based on very limited data. More reliable estimates are available for only two groups, the digeneans and monogeneans. Estimates were 2,270 digenean species in the 1,300 fish species of the GBR [4] and 2,000 monogenean species on the 1,000 fish species recorded around Heron Island, in the southern GBR [5].

An eight-year program allowed us to investigate the biodiversity of fish parasites off New Caledonia (South Pacific), the largest coral lagoon of the world. A compilation of available literature including a number of papers produced by this program [6] concluded that only 2% of fish parasite biodiversity was known in New Caledonia. Two subsequent comprehensive papers provided abundant, previously unpublished data and a compilation of already published information on two families of fish, the Serranidae (groupers) and the Lethrinidae (emperors) [7,8]. In this paper, we provide information about the parasites of the Lutjanidae, Nemipteridae and Caesionidae and compare our results with those already published for the other families.

## Results

Results are presented as a host-parasite list (Appendix 1), a parasite-host list (Appendix 2) and a list of material deposited (Appendix 3). The number of host-parasite combinations (HPCs) and the number of species-level identified parasite – host-parasite combinations (SLIP-HPCs) found in each fish species are given in Table 1.

**Table 1:** Number of host-parasite combinations (HPCs) found in 24 species of caesionids, lutjanids and nemipterids in New Caledonia

**Family and habitat**	**Fish species**	**Total**	**Gill**	**Abdo**	**Isop**	**Cope**	**Mono**	**Poly**	**Dige**	**Both**	**Tetr**	**Tryp**	**Nema**	**Other**	**Total**
Caesionidae	*Caesio cuning*	8	4	8					2(0)	1(0)	1(0)		1(0)		5(0)
Lutjanidae, reef-associated	*Aprion virescens*	3	3	3			1(1)		2(2)						3(3)
	*Lutjanus adetii*	5	0	5					2(2)	1(0)			1(0)		4(2)
	*Lutjanus argentimaculatus*	4	3	3	1(0)	2(1)	5(3)		4(4)		1(0)				13(8)
	*Lutjanus fulviflamma*	17	11	11		2(1)	3(2)	1(0)	2(2)				1(0)		9(5)
	*Lutjanus fulvus* *	2	1	0			4(2)								4(2)
	*Lutjanus gibbus* *	2	2	1		1(1)	1(0)								2(1)
	*Lutjanus kasmira*	16	12	12		1(0)	6(0)		5(2)	1(0)	1(0)		1(0)		15(2)
	*Lutjanus monostigma* *	0	0	0	1(1)										1(1)
	*Lutjanus quinquelineatus*	12	0	6			5(3)		2(2)		1(0)		1(0)		9(5)
	*Lutjanus rivulatus* *	2	2	2			1(0)						1(0)	1(0)	3(0)
	*Lutjanus russellii*	6	0	1	1(0)		4(2)	1(0)	2(1)						8(3)
	*Lutjanus vitta*	42	19	31		1(0)	6(2)		5(3)		1(0)	2(2)	5(2)		20(9)
	*Macolor niger* *	2	2	1		3(1)	2(0)		1(0)						6(1)
Lutjanidae, deep-sea	*Etelis carbunculus*	16	5	3	1(1)	4(1)	1(1)		3(2)		1(0)	1(0)	2(1)	1(0)	14(6)
	*Etelis coruscans*	18	11	5	1(1)	4(1)	2(2)	1(0)	3(1)			1(0)	2(2)		14(7)
	*Pristipomoides argyrogrammicus*	20	14	18	1(1)	1(0)	3(1)		4(3)		1(0)	1(0)	2(1)		13(6)
	*Pristipomoides auricilla* *	2	2	2	1(0)		1(0)		1(1)		1(0)	1(0)	3(1)		8(2)
	*Pristipomoides filamentosus*	7	2	2	2(2)	1(1)	1(0)		2(0)				3(2)		9(5)
Nemipteridae	*Nemipterus furcosus*	239	111	160	1(0)		2(0)	1(0)	5(4)	1(0)	1(0)	6(6)	7(3)	1(0)	25(13)
	*Pentapodus aureofasciatus*	23	19	12	1(0)	1(0)	2(1)	1(0)	4(0)		1(0)		2(0)		12(1)
	*Pentapodus nagasakiensis* *	2	2	2					2(0)		1(0)		1(0)		4(0)
	*Scolopsis bilineata*	12	8	9	1(0)				2(1)				1(0)	1(0)	5(1)
	*Scolopsis taenioptera* *	3	2	3									1(0)		1(0)
	Total Caesionidae (1 species)	8	4	8					2(0)	1(0)	1(0)		1(0)		5(0)
	Partial total Lutjanidae, reef (13)	113	55	76	3(1)	10(4)	38(15)	2(0)	25(18)	2(0)	4(0)	2(2)	10(2)	1(0)	97(42)
	Partial total Lutjanidae, deep-sea (5)	63	34	30	6(5)	10(3)	8(4)	1(0)	13(7)	0(0)	3(0)	4(0)	12(6)	1(0)	58(25)
	Total Lutjanidae (18 species)	176	89	106	9(6)	20(7)	46(19)	3(0)	38(25)	2(0)	7(0)	6(2)	22(8)	2(0)	155(67)
	Total Nemipteridae (5 species)	279	142	186	3(0)	1(0)	4(1)	2(0)	13(5)	1(0)	3(0)	6(6)	12(3)	2(0)	47(15)
	Total (24 species)	463	235	300	12(6)	21(7)	50(20)	5(0)	53(30)	4(0)	11(0)	12(8)	35(11)	4(0)	207(82)

*: species with low sample size or only anecdotal collections, excluded from general calculations in Table 3. For each number: HPCs (SLIP-HPCs) i.e. number of host-parasite combinations, and, within parentheses, number of species level identified parasite – host-parasite combinations.

## Discussion

### Comments on each group

For brevity, in this section references to our own published papers on these fish families (available in Table 2 and Appendix 2) are kept to a minimum. For parasites, the “minimized number of taxa” is a cautious minimized evaluation in which all unidentified taxa in a group are counted as a single taxon [8].

**Table 2:** List of 58 species identified at the species level with Latin binomial, with full authorities

	
**Isopoda (4)**	Aegidae: *Aega musorstom *Bruce, 2004
	Corallanidae: *Argathona macronema *(Bleeker, 1857) Monod, 1933
	Cymothoidae: *Anilocra gigantea* (Herklots, 1870) Schiœdte & Meinert, 1881
	Cymothoidae: *Anilocra longicauda* Schiœdte & Meinert, 1881
**Copepoda (6)**	Caligidae: *Caligus brevis* Shiino, 1954
	Dissonidae: *Dissonus excavatus* Boxshall, Lin, Ho, Ohtsuka, Venmathi Maran & Justine, 2008
	Hatschekiidae: *Hatschekia clava* Kabata, 1991
	Hatschekiidae: *Hatschekia tanysoma* Ho & Kim, 2001
	Lernaeopodidae: *Parabrachiella lutiani* (Pillai, 1968)
	Pennellidae: *Lernaeolophus sultanus* (H Milne Edwards, 1840) Heller, 1865
**Monopisthocotylea (11)**	Ancyrocephalidae: *Haliotrematoides lanx* Kritsky & Justine, 2009 in Kritsky, Yang & Sun, 2009
	Ancyrocephalidae: *Haliotrematoides longitubocirrus* (Bychowsky & Nagibina, 1971) Kritsky, Yang & Sun, 2009
	Ancyrocephalidae: *Haliotrematoides novaecaledoniae* Kritsky & Justine, 2009 in Kritsky, Yang & Sun, 2009
	Ancyrocephalidae: *Haliotrematoides patellacirrus* (Bychowsky & Nagibina, 1971) Kritsky, Yang & Sun, 2009
	Ancyrocephalidae: *Haliotrematoides potens* Kritsky & Justine, 2009 in Kritsky, Yang & Sun, 2009
	Ancyrocephalidae: *Haliotrematoides tainophallus* Kritsky & Justine, 2009 in Kritsky, Yang & Sun, 2009
	Capsalidae: *Benedenia elongata* (Yamaguti, 1968) Egorova, 1997
	Capsalidae: *Lagenivaginopseudobenedenia etelis* Yamaguti, 1966
	Capsalidae: *Pseudonitzschia uku* Yamaguti, 1965
	Capsalidae: *Trilobiodiscus lutiani* Bychowsky & Nagibina, 1967
	Diplectanidae: *Calydiscoides limae* Justine & Brena, 2009
**Digenea (21)**	Acanthocolpidae: *Pleorchis uku* Yamaguti, 1970
	Acanthocolpidae: *Stephanostomum uku* Yamaguti, 1970
	Cryptogonimidae: *Adlardia novaecaledoniae* Miller, Bray, Goiran, Justine & Cribb, 2009
	Cryptogonimidae: *Euryakaina manilensis* (Velasquez, 1961) Miller, Adlard, Bray, Justine, & Cribb, 2010
	Cryptogonimidae: *Euryakaina marina* (Hafeezullah & Siddiqi, 1970) Miller, Adlard, Bray, Justine, & Cribb, 2010
	Cryptogonimidae: *Metadena rooseveltiae* (Yamaguti, 1970) Miller & Cribb, 2008
	Cryptogonimidae: *Retrovarium manteri* Miller & Cribb, 2007
	Cryptogonimidae: *Retrovarium saccatum* (Manter, 1963) Miller & Cribb, 2007
	Cryptogonimidae: *Siphoderina hirastricta* (Manter, 1963) Miller & Cribb, 2008
	Cryptogonimidae: *Siphoderina ulaula* (Yamaguti, 1970) Miller & Cribb, 2008
	Cryptogonimidae: *Varialvus charadrus* Miller, Bray, Justine & Cribb, 2010
	Fellodistomatidae: *Tergestia magna* Korotaeva, 1972
	Hemiuridae: *Ectenurus trachuri* (Yamaguti, 1934) Yamaguti, 1970
	Lepocreadiidae: *Lepidapedoides kalikali* Yamaguti, 1970
	Monorchiidae: *Allobacciger macrorchis* Hafeezullah & Siddiqi, 1970
	Opecoelidae: *Hamacreadium mutabile* Linton, 1910
	Opecoelidae: *Macvicaria jagannathi* (Gupta & Singh, 1985) Bijukumar, 1997
	Opecoelidae: *Neolebouria blatta* Bray & Justine, 2009
	Opecoelidae: *Neolebouria lineatus* Aken’Ova & Cribb, 2001
	Sclerodistomidae: *Prosogonotrema bilabiatum* Vigueras, 1940
	Transversotrematidae: *Transversotrema borboleta* Hunter & Cribb, 2012
**Trypanorhyncha (7)**	Lacistorhynchidae: *Callitetrarhynchus gracilis* (Rudolphi, 1819) Pintner, 1931
	Lacistorhynchidae: *Floriceps minacanthus* Campbell et Beveridge, 1987
	Lacistorhynchidae: *Pseudolacistorhynchus heroniensis* (Sakanari, 1989) Palm, 2004
	Otobothriidae: *Otobothrium mugilis* Hiscock, 1954
	Tentaculariidae: *Nybelinia goreensis* Dollfus, 1960
	Tentaculariidae: *Nybelinia indica* Chandra, 1986
	Tentaculariidae: *Nybelinia queenslandensis* Jones & Beveridge, 1998
**Nematoda (9)**	Anisakidae: *Raphidascaris (Ichthyascaris) etelidis* Moravec & Justine, 2012
	Anisakidae: *Raphidascaris (Ichthyascaris) nemipteri* Moravec & Justine, 2005
	Camallanidae: *Camallanus carangis* Olsen, 1952
	Capillariidae: *Pseudocapillaria novaecaledoniensis* Moravec & Justine, 2010
	Cucullanidae: *Cucullanus bourdini* Petter & Le Bel, 1992
	Cucullanidae: *Dichelyne etelidis* Moravec & Justine, 2011
	Philometridae: *Philometra brevicollis* Moravec & Justine, 2011
	Philometridae: *Philometra mira* Moravec & Justine, 2011
	Trichosomoididae: *Huffmanela branchialis* Justine, 2004

This Table shows all binomial names of parasite taxa collected (SLIPs); since several names are extremely long, its main purpose is to lighten the other tables and the text. Authors involved in the description and combination of taxa for Isopoda: [28,29,32,167,168]; for Copepoda: [36,37,155,169-172]; for Monopisthocotylea: [45,49,66,67,156,173-175]; for Digenea: [71-74,76,77,79,84,86,87,89,97,176-180]; for Trypanorhyncha: [99,181-188]; for Nematoda: [24,157-161,189,190].

### Fish

In this paper, we group results from three families of fish, namely the Lutjanidae, Nemipteridae and Caesionidae. Clearly, most of the results concern the Lutjanidae but we included the two other families because they are closely related [9-11]. Modern molecular phylogenies are available for the Lutjanidae [12-15] and confirm the close relationship of the Lutjanidae and Caesionidae.

According to the most recent survey [16], the Lutjanidae, Caesionidae and Nemipteridae include, respectively, 17, 4, and 5 genera and 108, 22 and 66 species, with a total of 26 genera, 196 species. The numbers of species in New Caledonia [17] are, respectively, 43, 13, and 16, with a total 72 species. In this work, we report parasitological results from 18 lutjanid species, 1 caesionid and 6 nemipterids; the total, 25 species, represents 34% of the species reported from New Caledonia, and 13% of the world number of species for the three families.

Diets of lutjanids and nemipterids off New Caledonia mainly comprise fish, crustaceans and occasionally molluscs [18], all of which can serve as intermediate hosts for parasites such as nematodes, digeneans and cestodes.

Most fishes included in this study are reef-dwelling; however, we also include several lutjanids (two species of *Etelis* and three species of *Pristipomoides)* which are deeper water fishes, collected from the outer slope of the barrier reef of New Caledonia [19]. These fishes provide data for a comparison of the parasitic fauna of coral-associated and deeper sea fishes.

As occurs often in the South Pacific, parasitologists have had to face problems with fish taxonomy [8,20-23]. *Pentapodus aureofasciatus* Russell, 2001, was first identified as *Pentapodus* sp. in the description of a nematode [24] but this was corrected later [25].

### Isopoda

Adult isopods were rare and belonged to three families: Aegidae, Corallanidae and Cymothoidae. The single aegid, *Aega musorstom*, was found on a deep water lutjanid. Two cymothoids (*Anilocra gigantea* and *An. longicauda*) were found only on deep water lutjanids, but the single corallanid, *Argathona macronema*, was found on a coral dwelling lutjanid.

**Figure 1:**
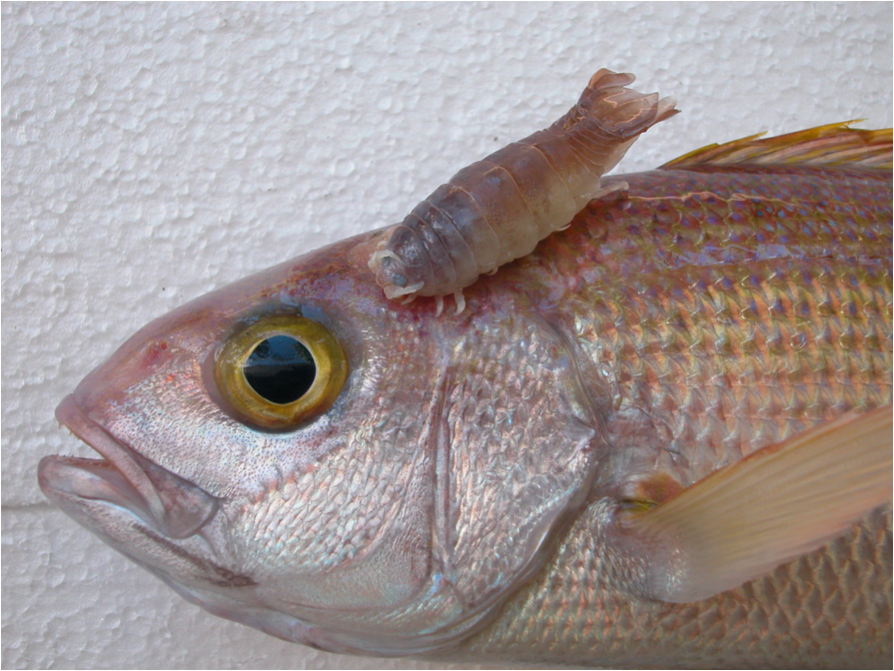
***Anilocra gigantea *(Isopoda, Cymothoidae), specimen MNHN Is6292, on the deep-sea lutjanid *Pristipomoides filamentosus*.**

*An. gigantea* was already known from New Caledonia and was recorded from the branchial region of the deep water lutjanid *Etelis carbunculus* off “Banc de la Torche, au sud-est de la Nouvelle Calédonie” [26]. It was also recorded from the Pacific Ocean from the gills of *Epinephelus* sp. and *Pr. flavipinnis*, off Suva reefs, Suva, Fiji [27] and from the Indian Ocean from an unidentified host [28-30]. We found this species again on *Et. carbunculus*, but *Et. coruscans* and *Pr. filamentosus* are new host records. Interestingly, we did not collect this species from the branchial region or from the gills of the host fish, as reported by previous authors but on the anterior part of the body just behind the head. A female specimen of *An. gigantea* attached behind the head of *Pr. filamentosus* is illustrated by a colour photograph (Figure 1).

*An. longicauda* was already known from the Indian and the Pacific Oceans [30]. It was previously recorded from Swains Reefs, Great Barrier Reef, Marion Reef, Australian Coral Sea, North West Shelf of Western Australia, Krakatua, Indonesia [31], Singapore and Poulo Condor, Vietnam [29,31], Ragay Gulf, Pasacao and Maribuyoc Bay, Bohol Island, Philippines [27]. This species has been reported from *Plectorhynchus goldmani*, *Diagramma picta* and *Priacanthus* sp. [31]. *Pr. argyrogrammicus* is a new host record and New Caledonia is a new geographical record.

*Aega musorstom* was already known from New Caledonia, in the vicinity of Western New Caledonia including the Coral Sea region of the Chesterfield Archipelago and the Loyalty Islands, at depths from 475 to 615 m [32]. Only one fish association was noted, “*Synagonopi* sp. 1” probably a species of *Synagrops* (Acropomatidae) [32]. *Pr. filamentosus* is a new host record.

*Argathona macronema* was already known from New Caledonia [7,33]. It was previously reported from *Epinephelus tauvina*, *Diagramma cinerascens*, *Pseudolabrus* sp., *Trachichtodes affinis*, *Cromileptes altivelis*, *Lu. argentimaculatus*, *Plectropomus leopardus* and *Pl. maculatus*[33]. It was found again later on *Pl. leopardus* and in addition on *Pl. laevis*[7]. *Lu. monostigma* is a new host record.

Larval isopods belonged to the Gnathiidae. Gnathiids, found as praniza larvae, were collected on 6 species of nemipterids and lutjanids (5 reef-dwelling, 1 deep water). In New Caledonia, larval gnathiids were found on most fish families examined (serranids, lethrinids, lutjanids, nemipterids and many others). Adult isopods were found on serranids and lutjanids but not on lethrinids and nemipterids [7,8]. The biodiversity of larval gnathiids is hard to evaluate [34,35], but it is likely that several species are involved.

### Copepoda

**Figure 2:**
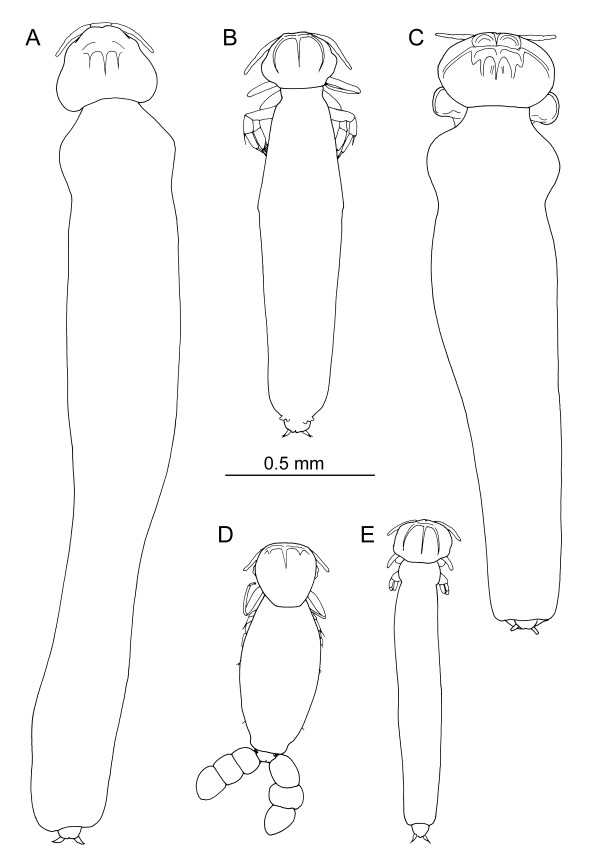
**Undescribed new species of *Hatschekia *(Copepoda, Hatschekiidae) collected from lutjanid hosts off New Caledonia, all drawn to same scale.** A, *Hatschekia* new species 21; B, *Hatschekia* new species 18; C, *Hatschekia* new species 20; D, *Hatschekia* new species 17; E, *Hatschekia* new species 19.

Fourteen taxa, including 6 identified at the species level, were found. Seven species of *Hatschekia* were distinguished but only two are known species, the other five (Figure 2) are not formally described. A total of 21 undescribed *Hatschekia* species has now been listed from New Caledonian fish ( [7,8]; this paper). *Hatschekia tanysoma* was originally described from Kuwait Bay, from *Lu. fulviflamma*[36] and is reported here from the Pacific for the first time, but from the same host. In contrast *H. clava* was described from Heron Island from material collected from *Lu. carponotatus* (Richardson) (as *Lu. chrysotaenia*) [37].

**Figure 3:**
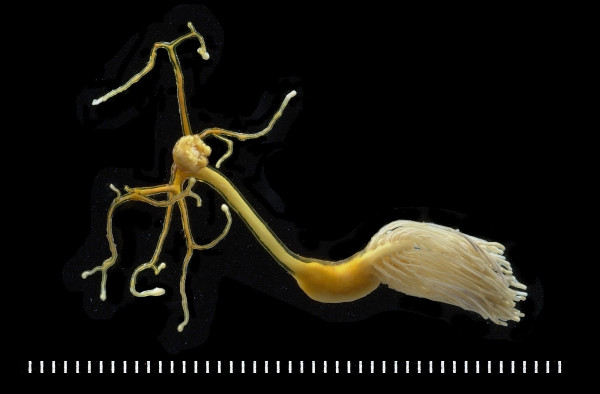
***Lernaeolophus sultanus *(Copepoda, Pennellidae), specimen BMNH 2010.750, from *Pristipomoides filamentosus *off New Caledonia. **Scale, each scale division = 1 mm.

The copepods belonged to five families, namely Caligidae, Dissonidae, Hatschekiidae, Lernaeopodidae and Pennellidae. Larvae and premetamorphic adults belonging to the Pennellidae were found on the deep-sea lutjanid *Et. coruscans*. The only adult member of this family found during eight years of sampling was a single female of *Lernaeolophus sultanus* (Figure 3) found on *Pr. filamentosus*. Pennellids are known to utilise two different hosts during their life cycle, either two different fish hosts or a pelagic mollusc and a final fish host [38]. However, the life cycle of no *Lernaeolophus* species has ever been elucidated so it is not possible to confirm whether the developmental stages found on *Pr. filamentosus* are those of *L. sultanus*. *L. sultanus* exhibits the lowest host specificity of any copepod parasite, occurring on 16 different host fishes in the Mediterranean [39].

*Caligus brevis* is reported here from two species of *Etelis*, *Et. carbunculus* and *Et. coruscans*, for the first time. This species was previously reported only from labrid hosts in Japanese [40] and New Zealand waters [41]. Ho & Lin (2004) suspected that *C. brevis* might be synonym of *Caligus oviceps* Shiino, 1952, but refrained from synonymizing them [42]. *C. oviceps* has already been reported from a lethrinid host (*Lethrinus haematopterus* Temminck & Schlegel) but it has a broad range of hosts including species of Cheilodactylidae, Kyphosidae, Monacanthidae, Mullidae, Scaridae, and Siganidae [42].

All the copepods are from the gills; none was found on the skin. Insufficient sampling precludes interpretation of the absence of copepods from several of the fish species listed here; however, the absence of copepods on fork-tailed threadfin breams, *Ne. furcosus*, with 239 specimens examined at various seasons during eight years is certainly significant.

### Monogenea

The minimized number of taxa for polyopisthocotyleans was 2 and for monopisthocotyleans was 23.

Polyopisthocotyleans were represented by *Allomicrocotyla* sp. on the deep-sea lutjanid *Et. coruscans*, and several records of unidentified microcotylids or other polyopisthocotylean families from coral-associated lutjanids and nemipterids. Polyopisthocotyleans on reef lutjanids were rare; similarly, polyopisthocotyleans were rare on *Lu. griseus* in the Gulf of Mexico [43]. The rarity of polyopisthocotyleans from 4 species of lutjanids off Heron Island on the Great Barrier Reef is apparent from the literature [44] and from unpublished observations by I.D. Whittington at the same locality.

Monopisthocotyleans included four families, the Ancyrocephalidae, Capsalidae, Diplectanidae and Gyrodactylidae.

Ancyrocephalids included a series of species of the recently described genus *Haliotrematoides* Kritsky, Yang & Sun, 2009, several species of *Euryhaliotrema* Kritsky & Boeger, 2002, which are still undescribed, and we also have records of various ancyrocephalids not attributed to a genus. Clearly, the lutjanids harbour an impressive ancyrocephalid radiation, with probably several species on each fish species; some of these species seem to be strictly species-specific, others are found on up to 5 different host species [45]. These ancyrocephalids might be a threat for cultured snappers [46].

Diplectanids included several species of *Diplectanum* and one species of *Calydiscoides*. *Calydiscoides* species are numerous in lethrinids [8]; only one species was found in New Caledonia in the families studied here, in the nemipterid *Pentapodus aureofasciatus*, but none in members of *Scolopsis* and *Nemipterus*, which are known to harbour sometimes numerous species of *Calydiscoides* in other localities [44,47,48].

Four species are attributed here to *Diplectanum*. *D. opakapaka* Yamaguti, 1968 was described from *Pr. microlepis* and *Aphareus rutilans* off Hawaii [49] and *D. curvivagina* Yamaguti, 1968 was described from *Pr. sieboldii* and *Pr. auricilla* off Hawaii [49] and have rarely been recorded since [50]. *D. fusiformis* Oliver & Paperna, 1984, described from *Lu. kasmira* off Kenya [51] was recorded again from its type-host and *Lu. fulvus* in French Polynesia and Hawaii [52,53]. *D. spirale* Nagibina, 1976 described from *Lu. fulviflamma*[54] has apparently not been recorded since its original description. In the absence of a comparative examination of the type specimens, we prefer to keep our identifications of these four species as “cf.” and we do not comment on their generic attribution.

Gyrodactylids were represented by a single specimen found on *Macolor niger*, which is a relatively difficult fish to catch and unfortunately additional specimens could not be obtained. It is the first gyrodactylid collected from a lutjanid. It should be noted that several coral-associated lutjanids were soaked for collection of skin monogeneans such as capsalids and digeneans such as transversotrematids, but no other gyrodactylid specimens were recovered.

Capsalid systematics is currently under reinvestigation (e.g. [55-57]) after Perkins et al. [58] demonstrated that the current morphological classification has limited congruence with a phylogenetic hypothesis based on three unlinked nuclear genes. Apparently homoplastic morphological features were highlighted throughout the molecular phylogeny [58]. This has entailed a reluctance to assign some taxa to genera until appropriate characters and generic and subfamilial definitions are better refined within a phylogenetic context. Hence ‘identifications’ of four taxa are herein provided only as Capsalidae sp. 6, 7, 13 and 17, differentiated phylogenetically by Perkins [59] using nuclear and mitochondrial markers, but from morphological ‘identifications’ made by I.D. Whittington.

Of the Capsalidae we report here, four from the gills were identified to species (*Benedenia elongata* from three lutjanid species; *Lagenivaginopseudobenedenia etelis* from *Et. coruscans*; *Pseudonitzschia uku* from *Aprion virescens*; *Trilobiodiscus lutiani* from *Lu. argentimaculatus*). Two capsalid species recovered from gills of *Macolor niger* and *Ne. furcosus* remained unidentified. A species assigned only as a *Metabenedeniella* sp. was recovered from the pectoral fins (see [59]; probably a new species, I.D. Whittington unpublished) and the four capsalid species assigned only as Capsalidae sp. 6, 7, 13 and 17 [59] were recovered from the gills, fins, body washings, branchiostegal membranes and the head of their hosts (for details, see Appendix 1). Additional external sites, rarely examined in this study, received careful scrutiny only when I.D. Whittington visited Nouméa in October/November 2008. During his visit, thorough fish necropsies (four specimens of *Lu. vitta*; six specimens of *Ne. furcosus*; one specimen of *Lu. kasmira* [which was totally uninfected by capsalids]; two specimens of *Pr. argyrogrammicus*; one specimen of *Lu. argentimaculatus*) paid particular attention to external microhabitats known to support capsalids (e.g. [60]) followed by freshwater bathing of the same tissues to ensure recovery of parasites that may be cryptic due to camouflage or transparency (e.g. [61]). Dissections of fish at other times, when microhabitats other than gills that may harbour capsalids remained unstudied, seem likely to have appreciably underestimated the diversity of capsalid monogeneans from the caesionids, lutjanids and nemipterids examined.

Other factors in this study that contributed to an inability to assign capsalids to genera from gills, pelvic, pectoral and anal fins, body washings, branchiostegal membranes and head included small numbers of specimens recovered and the juvenile status of many capsalid individuals from *Ne. furcosus*. Adult specimens of *B. lutjani* from *Lu. carponotatus* from Heron Island on the Great Barrier Reef preferentially inhabit the branchiostegal membranes, and the pelvic fins was the site where protandrous parasites that possess a vagina may become inseminated [62,63]. Discovery of juvenile specimens of Capsalidae sp. 13 on pelvic and anal fins and branchiostegal membranes and small, recently matured adults on the branchiostegal membranes of *Ne. furcosus* suggests a similar migration and habitat partitioning for this taxon on the nemipterid.

Several of the capsalids reported in this study represent new records. *Metabenedeniella* species are previously reported from oplegnathids, serranids and haemulids [64]. Our report of a *Metabenedeniella* sp. from a lutjanid (Appendix 1) represents a new fish family and a new geographic record for this capsalid genus. *Benedenia elongata* was described as *Pseudobenedenia elongata* from a priacanthid, *Priacanthus boops*, and two lutjanids, *Pr. sieboldii* and *Arnillo auricilla* (now *Pr. auricilla*, see [65]), off Hawaii [49]. Although two specimens of *Pr. auricilla* were studied in the present investigation, *B. elongata* was not recorded. It has, however, been recorded from three new host lutjanid species, *Et. carbunculus*, *Et. coruscans* and *Pr. argyrogrammicus*, each of which represents a new host and geographic record for *B. elongata*. Like deep water lutjanids, priacanthids can also occur in deep water and it seems as though *B. elongata* has relatively low host specificity among several deeper water fish species in these two families.

*Lagenivaginopseudobenedenia etelis* was originally described from *Et. carbunculus* off Hawaii by Yamaguti [66]. While 16 specimens of *Et. carbunculus* from New Caledonia were studied, *La. etelis* was not reported but we did record it from *Et*. *coruscans*, a new host and geographic record for this taxon (Appendix 1). As suggested for *B. elongata*, *La. etelis* may also exhibit relatively low host specificity and infect several deep-sea lutjanids. Further sampling may indicate whether *La. etelis* is specific to species of *Etelis* or whether this capsalid can also infect *Pristipomoides* species.

As far as we are aware, there have been no published reports of *Trilobiodiscus lutiani* since its original description [67] although it does occur on the type host, *Lu. argentimaculatus* in north Queensland (I.D. Whittington, unpublished). The present report of *T. lutiani* from New Caledonia is a new geographic record (Appendix 1).

For the same sampling limitations presented above, it is possible that specimens of Anoplodiscidae known from external surfaces of nemipterids on the Great Barrier Reef ( [68]; I.D. Whittington, unpublished) may have been overlooked in the present study.

### Digenea

The total minimized number of taxa was 33, with 21 species identified at the species level (SLIPs).

Eleven families were represented: Acanthocolpidae (2 SLIPs), Cryptogonimidae (12 spp, 9 SLIPs), Didymozoidae (2 unidentified adults, unknown number of species as unidentified larvae), Fellodistomatidae (1 SLIP), Hemiuridae (3 spp, 1 SLIP), Lecithasteridae (1 sp.), Lepocreadiidae (1 SLIP), Monorchiidae (1 SLIP), Opecoelidae (8 spp, 4 SLIPs), Sclerodistomidae (1 SLIP) and Transversotrematidae (1 SLIP).

The dominant digenean family is the Cryptogonimidae. Members of this family and of the Acanthocolpidae and the Didymozoidae utilise fishes as second intermediate hosts [69], indicating that this component of the lutjanid (and related families) diet is a major source of its digenean fauna. The other digenean families utilise a wide range of invertebrates, often crustaceans, as second intermediate hosts, and occasionally lepocreadiids and opecoelids also use fishes [69]. Some hemiuroids (e.g. *Lecithochirium*) have interpolated a third intermediate host, a fish, into their life-cycle [70]. Considering the importance of fishes in the diet and infection of lutjanids it is, perhaps, surprising that we found no members of the common family Bucephalidae, which also utilises fishes as second intermediate hosts.

The cryptogonimids include one species, *Adlardia novaecaledoniae*, which is known only from New Caledonia [71]. Other identified cryptogonimids are reported more widely, from the Great Barrier Reef (GBR) (*Retrovarium manteri*), from Fiji (*R. saccatum*), from both these localities (*Siphoderina hirastricta*), from Hawaii (*Metadena rooseveltiae*), from Hawaii and China (*S. ulaula*) and from the Philippines and the GBR (*Euryakaina manilensis*) [72-76]. Two species also occur in the Indian Ocean. *Varialvus charadrus* occurs in the GBR and the Maldives and *E. marina* is reported from the GBR, the Bay of Bengal and Ningaloo Reef, Western Australia [77-79].

Both acanthocolpid species are reported from *Aprion virescens*, and are known from the Western Pacific, from Hawaii to the Great Barrier Reef [74,75,80,81].

The Opecoelidae is a difficult group, with many similar species described. Of the four SLIPs one, *Hamacreadium mutabile*, is a cosmopolitan parasite reported in many lutjanid species in the Atlantic, Indian and Pacific Oceans [82]. This is one of the few opecoelids known to utilise fishes as its second intermediate host [83]. Of the other species, *Neolebouria blatta* is reported only from New Caledonia, *N. lineatus* only from southern Western Australia, and *Macvicaria jagannathi* from the Bay of Bengal [84-88].

The Transversotrematidae includes a single species, which could be identified at the species level. Morphologically and biologically this form agrees with the variable species *Transversotrema borboleta*, reported from chaetodontids and lutjanids (including *Lu. kasmira*) from the northern and southern GBR [89]. The species includes 3 genotypes which are not partitioned to different host families, but only genotype ‘G2’ is reported in *Lu. kasmira*.

Didymozoidae include several records of unidentified juveniles from coral and deep-sea lutjanids and nemipterids; juvenile didymozoids are found in the intestine of most marine tropical fish [7,8,90,91] and the present records are not surprising. Adult didymozoids include a relatively abundant long filiform form found under the scales of the deep water *Et. carbunculus*.

Two of the other SLIPs are widespread in the Atlantic and Indo-West Pacific Region, i.e., *Ectenurus trachuri*, *Prosogonotrema bilabiatum* (see [92,93]). *E. trachuri* is mostly reported in carangids, but *P. bilabiatum* is a common parasite of lutjanids. *Allobacciger macrorchis* (also known as *Monorcheides macrorchis*) is reported mainly in the Indian Ocean, but also Japan [77,94,95]. *Lepidapedoides kalikali* is known only from Hawaii, Japan and Palau [74,96]. *Tergestia magna* is reported from emmelichthyid fishes from the waters off southern Australia [97].

### Cestoda Bothriocephalidea and Tetraphyllidea

For these two cestode orders, only larvae were found, and species identification is not possible.

Bothriocephalideans were represented by larvae found in four species of fishes. They were especially abundant and highly prevalent in *Ne. furcosus*. White, flattish larvae, about 1 cm in length, were found in the abdominal cavity and in almost all organs of this fish, including the intestinal lumen. Sometimes larvae were visible when the fish was caught, because they were protruding in the region around the anus, with half of their body buried in the flesh. We interpret these larvae as bothriocephalideans due to the lack of morphological characters.

Tetraphyllideans include small forms found in the intestinal lumen of various fishes, both from coral-associated and deeper water fishes. It is possible that these include lecanicephalideans. A detailed morphological examination was not performed.

### Cestoda Trypanorhyncha

Only larvae were found. All these trypanorhynchs have their adults parasitic in sharks and are probably transmitted to their final host by predation. Seven species of larval trypanorhynch cestodes, all from coral-associated fish, were identified at the species level on the basis of the armature of their tentacles.

Trypanoselachoidans [98] included four species, found as larvae in cysts in the abdominal cavity. The otobothriid *Otobothrium mugilis* was found only once in the nemipterid *Nemipterus furcosus*. The lacistorhynchids *Callitetrarhynchus gracilis, Floriceps minacanthus* and *Pseudolacistorhynchus heroniensis* were found in a lutjanid and a nemipterid; in New Caledonia, *C. gracilis* has already been recorded from 4 serranids and 1 lethrinid, *F. minacanthus* from 6 serranids and 1 lethrinid, and *Ps. heroniensis* from 7 serranids and 2 lethrinids [7,8] and show low host specificity at the larval stage. *C. gracilis* has already been found in *Lu. vitta* in Indonesia and *F. minacanthus* has already been found in *Ne. furcosus* in Indonesia [99]. *Ne. furcosus* is a new host record for *C. gracilis*, *Lu. vitta* is a new host record for *Ps. heroniensis* and *Ne. furcosus* is a new geographical and new host record for *O. mugilis*.

Trypanobatoidans [98] included three species of the tentaculariid genus *Nybelinia*, found free in the intestinal lumen of the nemipterid *Ne. furcosus*. *Ny. goreensis* has already been found in *Ne. furcosus* in Indonesia [99] and in New Caledonia in 2 lethrinids [8]. *Ne. furcosus* is a new geographical and new host record for *Ny. indica* and for *Ny. queenslandensis.*

**Figure 4:**
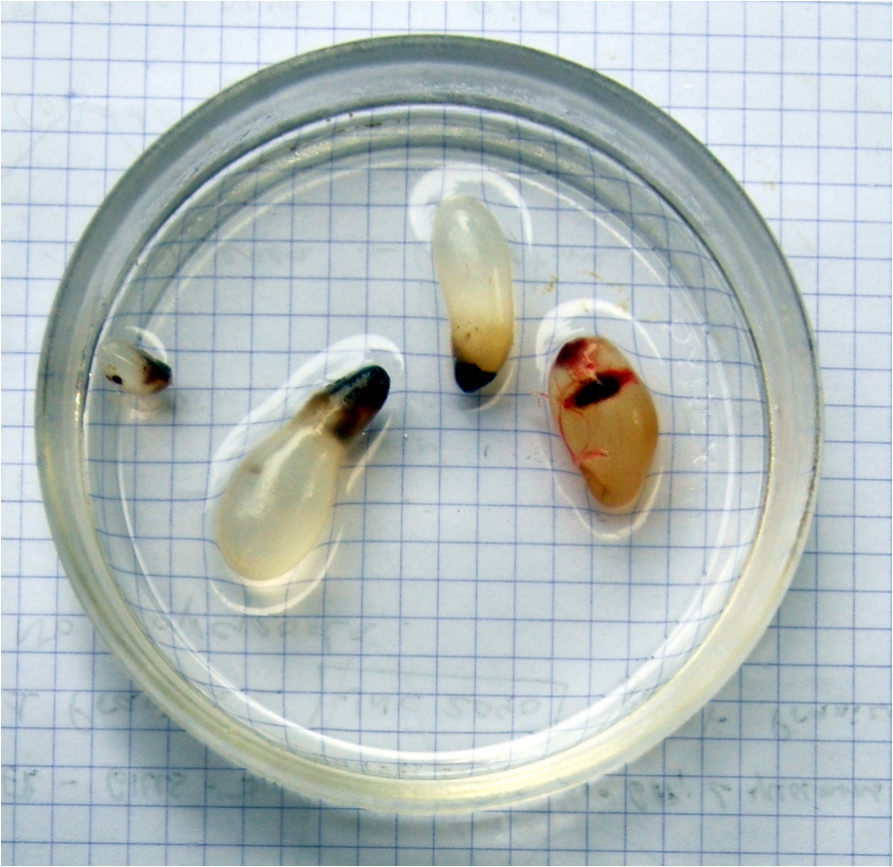
**Sterile cysts from deep-sea lutjanids (here from *Pristipomoides auricilla*), interpreted as immature cysts of trypanorhynch cestodes. **Scale, each square = 5 mm.

It is likely that this high rate of records in *Ne. furcosus* is simply a consequence of its higher sampling (see also nematodes). In addition, cysts were repeatedly found in small numbers in the abdominal cavity of several deep water lutjanids. Although there is no direct evidence that these represent trypanorhynchs because they never contained larvae, the cysts (Figure 4), about 1 cm in length, were similar to sterile trypanorhynch cysts. We hypothesize that these larvae have a very long development time, perhaps related to the relatively cold deep water environment, and that the fish collected by us were too young to harbour fully developed larvae; deep water lutjanids have long life spans (30–40 years) [100].

### Nematoda

The minimized number of taxa is 17, including 9 species identified at the species level.

Nematodes recorded belonged to eight families, the Anisakidae, Camallanidae, Capillariidae, Cucullanidae, Gnathostomatidae, Philometridae, Physalopteridae and Trichosomoididae.

The Anisakidae is represented by both larvae and adults. Larvae were encapsulated on the surface of organs or free in the lumen of the intestine; a few were occasionally identified at the genus level (*Anisakis* sp., *Hysterothylacium* sp., *Terranova* sp.), but most were identified only at the family level. Most fish species, whether coral-associated [7,8] or deep-sea, harbour these larvae, sometimes in very high numbers.

Adult anisakids included two newly described species of *Raphidascaris (Ichthyascaris)*, one from the coral-associated nemipterid *Ne. furcosus*, and one from the deep water lutjanid *E. coruscans*, and several additional specimens were found in other fish species and could not be identified at the species level.

Camallanids included one identified species, *Camallanus carangis*, and unidentified immature specimens.

Cucullanids were found only in deep water lutjanids, with *Cucullanus bourdini* in two species of *Pristipomoides*, and *Dichelyne etelidis* in two species of *Etelis*.

Unidentified gnathostomatids and physalopterids were occasionally found only in *Ne. furcosus* and probably illustrate the fact that this fish species was more intensively sampled that others, thus providing a number of records of parasites with low prevalence (see also trypanorhynchs).

Gonad-parasitic philometrids were found as two new species from *Lu. vitta*, but never in other fish species. Lutjanids and nemipterids are known as hosts of a few species of gonad-parasitic philometrids in other regions, such as the North Pacific, Indian and Atlantic Oceans [101,102].

Capillariids included a newly described species, *Pseudocapillaria novaecaledoniensis*, from a deep water lutjanid, but no other records were made.

Trichosomoidids included two species of *Huffmanela*, *H. branchialis* and *Huffmanela* sp., from the gills of two nemipterids. Interestingly, both species recorded in 2004, were never found again, despite intensive sampling of *Ne. furcosus*; their prevalence is probably very low, and their initial discovery in a small sample should be attributed to chance. Similarly, no *Huffmanela* species was recorded from more than 500 serranids examined [7], but a new species was described later from a serranid [103]. Tissue-dwelling trichosomoidids are characterised by two opposing features which probably balance each other out - very low prevalences and extremely high numbers (millions) of eggs in the few individual fish infected.

### Hirudinea, ‘Turbellaria’ and Acanthocephala

Specimens of these three groups were rarely found.

Juvenile specimens of the piscicolid leech *Trachelobdella* sp. were found on the gills of a lutjanid and a nemipterid. Leeches of this genus are known from other lutjanids [104,105].

Cysts containing an unknown turbellarian were found rarely on the gills of *Ne. furcosus*; these were orange, abundant but with a very low prevalence, and were not studied in detail. Parasitic turbellarians are rarely found on coral reef fish [106,107].

An unidentified acanthocephalan was found once in the intestine of the deep water *Et. carbunculus*; this record constitutes a small addition to our very poor knowledge of the acanthocephalans of New Caledonia fish [6-8,108].

### A numerical evaluation of parasite biodiversity in lutjanids and nemipterids

In presenting our results, we used the same methods as in previous similar papers of this series on serranids and lethrinids [7,8].

Our results (Appendices 1 and 2) include a number of parasite identifications, but the level of taxonomic identification varies greatly. Table 1 details the number of HPCs found in each fish species, and indicates how many fish specimens were examined; it was compiled by counting each parasitological record (i.e. each line in Appendix 1) as a host-parasite combination (HPC).

The number of HPCs differs from the actual number of different parasite species, for two reasons (a) a parasite species present in several hosts is counted as several HPCs; and (b) HPCs in Table 1 enumerate records which vary widely in systematic precision, and may designate, in a decreasing order of taxonomic precision:

Species-level identified parasites (SLIPs); these have full binomial names, and we do not include ‘cf’ identifications within them.

Species-level identified parasites which have not yet received a binomial (such as the numbered copepods *Hatschekia* sp. 17–23, *Diplectanum* ‘cf.’ species, and numbered capsalids). These represent valid, independent species but a comparison of the presence of these species in other localities or in other fish species will not be possible until the parasite species are formally identified, described and names are published.

Parasite species identified at the generic level only, but which probably represent only a single species (examples: several digeneans).

Parasite species identified at the generic level only, but for which we already know that they represent several species (example: several ancyrocephalid monogeneans).

Parasite species identified at the family or higher level, for which we know that abundant biodiversity is hidden within this HPC. This includes unidentifiable larvae such as gnathiid isopods, anisakid nematodes, didymozoid digeneans and tetraphyllidean metacestodes. We estimate that these may represent a total of about 50–100 species.

Only species-level identified parasites with binomial (SLIPs) allow valid comparisons between localities and fish.

Table 1 includes all results, but some fishes were only studied superficially and their inclusion in further calculations would severely bias the results; for this reason, Table 3 was constructed only from fish species of which at least several specimens have been studied. Table 3 also provides a comparison with the lethrinids and serranids of New Caledonia, based on previous results [7,8]; this comparison will be discussed below. Of course, we are aware that the number of fish studied is generally too low to provide truly significant results on parasite biodiversity, but at least these results allow comparison with other fish families studied using the same methods in the same area. Caesionids are not included in Table 3 because only a single species was involved.

**Table 3:** Parasite biodiversity in lutjanids and nemipterids in New Caledonia for each parasite group, and a comparison with lethrinids and serranids

**Family or group**		**Isop**	**Cope**	**Mono**	**Poly**	**Dige**	**Both**	**Tetr**	**Tryp**	**Nema**	**Other**	**Total**
**Lutjanidae, reef-associated**	**HPCs**	2	6	30	2	24	2	4	2	9	0	**81**
(species: 8*; specimens: 105; gill: 48; abdomen: 72)	**SLIPs**	0	2	8	0	11	0	0	2	2	0	**25**
**Expressed as mean per fish species:**	**HPCs mean**	0.25	0.75	3.75	0.25	3.00	0.25	0.50	0.25	1.13	0.00	**10.13**
	**SLIPs mean**	0.00	0.25	1.00	0.00	1.25	0.00	0.00	0.25	0.25	0.00	**3.13**
**Lutjanidae, deep-sea**	**HPCs**	5	10	7	1	12	0	2	3	9	1	**50**
(species: 4*; specimens: 61; gill: 32; abdomen: 28)	**SLIPs**	3	2	2	0	4	0	0	0	4	0	**15**
**Expressed as mean per fish species:**	**HPCs mean**	1.25	2.50	1.75	0.25	3.00	0.00	0.50	0.75	2.25	0.25	**12.50**
	**SLIPs mean**	0.75	0.50	0.50	0.00	1.00	0.00	0.00	0.00	1.00	0.00	**3.75**
**Lutjanidae, all**	**HPCs**	7	16	37	3	36	2	6	5	18	1	**131**
(species: 12*; specimens: 166; gill: 80; abdomen: 100)	**SLIPs**	3	4	10	0	15	0	0	2	6	0	**40**
**Expressed as mean per fish species:**	**HPCs mean**	0.58	1.33	3.08	0.25	3.00	0.17	0.50	0.42	1.50	0.08	**10.92**
	**SLIPs mean**	0.33	0.33	0.83	0.00	1.25	0.00	0.00	0.17	0.50	0.00	**3.33**
**Nemipteridae**	**HPCs**	3	1	4	2	11	1	2	6	10	2	**42**
(species: 3*; specimens: 274; gill: 138; abdomen: 181)	**SLIPs**	0	0	1	0	5	0	0	6	3	0	**15**
**Expressed as mean per fish species:**	**HPCs mean**	1.00	0.33	1.33	0.67	3.67	0.33	0.67	2.00	3.33	0.67	**14.00**
	**SLIPs mean**	0.00	0.00	0.33	0.00	1.67	0.00	0.00	2.00	1.00	0.00	**5.00**
**Lethrinidae**	**HPCs**	9	21	53	4	59	1	7	10	21	3	**188**
(17 species: 17; specimens: 423; gill: 227; abdomen: 334)	**SLIPs**	0	7	11	0	13	0	0	6	5	0	**42**
**Expressed as mean per fish species:**	**HPCs mean**	0.53	1.24	3.12	0.24	3.47	0.06	0.41	0.59	1.24	0.18	**11.06**
	**SLIPs mean**	0.00	0.41	0.65	0.00	0.76	0.00	0.00	0.35	0.29	0.00	**2.47**
**Serranidae**	**HPCs**	20	53	97	0	76	4	13	35	37	2	**337**
(28 species, specimens: 540; gill: 394; abdomen: 275)	**SLIPs**	3	7	42	0	13	1	0	5	4	0	**75**
**Expressed as mean per fish species:**	**HPCs mean**	0.71	1.89	3.46	0.00	2.71	0.14	0.46	1.25	1.32	0.07	**12.04**
	**SLIPs mean**	0.11	0.25	1.50	0.00	0.46	0.04	0.00	0.18	0.14	0.00	**2.68**

HPCs, number of host-parasite combinations; SLIPs: number of species-level identified parasites (the same parasite species found in different hosts is counted a single time; hence differences with numbers of SLIP-HPCs in Table 1). Source of data for lutjanids and nemipterids: Table 1 and Appendix 2, this paper; for lethrinids and serranids, Table 3 in [8].

*: species with only anecdotal data (see Table 1) were excluded from these calculations.

For lutjanids (Table 3), the total number of HPCs was 131, and the number of different parasite species identified at the species level (SLIPs) was 40. For nemipterids (Table 3), the total number of HPCs was 42, and the number of SLIPs was 15. As usual, in addition, indistinguishable larval taxa probably correspond to a high number (50–100?) of different species, but cannot be differentiated on the basis of morphological studies.

Table 3 includes evaluations of these numbers as means per species of fish. The main results for all lutjanids (reef-associated and deeper sea) were that 10.92 HPCs were found per fish species, with 3.33 SLIPs (identified with binomial) per fish species. For nemipterids, the results were 14.00 HPCs and 5.00 SLIPs per fish species.

### A comparison between reef-associated and deeper-water lutjanids

Our results provide an opportunity to compare parasite biodiversity in reef-associated and deep water fishes. It is widely accepted that fishes in deeper waters have a lower parasitic diversity than surface fishes [109-112]. However, in comparative studies, fish species from the deep-sea are generally from orders (e.g. Gadiformes, Ophidiiformes, Notacanthiformes) which are different from the orders of surface fishes (e.g. Perciformes); in contrast, our study allows us to compare fish from the same family, the Lutjanidae, from both environments. Moreover, collection areas were very close and adjacent, with deeper-water fishes collected from just off the barrier reef along the outer slope, i.e. less than one kilometre away from the barrier reef and the lagoon [19]. Recently, a molecular study demonstrated that monogeneans of groupers tend to widen specificity when they infect fish from the outer slope, in comparison to lagoon fish where they are strictly specific [23].

Table 3 shows that the number of HPCs in reef-associated lutjanids was 10.13 per fish species, compared with 12.50 (123%) in deep-water lutjanids. The number of SLIPs in reef-associated lutjanids was 3.13 per fish species, compared with 3.75 (125%) in deeper water lutjanids. These figures suggest that parasite biodiversity was higher in deeper-water fishes than in reef-associated fishes, a highly unexpected result.

However, we identify four biases which diminish the validity of this comparison:

(a) Depth bias. Estimates of parasite biodiversity in deep-sea fish [109-112] generally concern fish from deeper seas (i.e. 1000 m vs 100–250 m) and from other fish orders than those studied here.

(b) Size bias. Most deeper water fish examined, especially *Eteli*s spp., were large fish, usually in the metre range size, while reef lutjanids were smaller, usually 10–30 cm in length [113]. It is known that parasite biodiversity increases with the size of hosts [8,114,115].

(c) Monogenean bias. Coral-associated lutjanids harbour a number of small ancyrocephalid monogeneans, of which a large proportion has not been described yet [45].

(d) Human bias. The high number of parasites identified at the species level in deeper water fish is probably related to the scientific interest they elicit in scientists. Systematicians like to describe rare parasites from rarely examined fish! No particular direction was given to participants of this study to balance their descriptive effort between reef and deeper water fish.

### A comparison with lethrinids and serranids

Data on parasite biodiversity, compiled using the same methods at the same location, are available for two other families [7,8].

Table 4 compares parasite biodiversity in four families of reef-associated fishes, the lutjanids and nemipterids (present study), and the serranids and lethrinids [7,8]. Table 4 also compares results for data compiled from fishes with variable sample size (anecdotal collections excluded) and for fishes with significant sampling (i.e. >25 individuals). For the latter, the numbers of HPCs for lutjanids and nemipterids per fish species are 20.00 and 25.00, respectively, and the number of SLIPs are 9.00 and 13.00, respectively. These results are similar to previous results in serranids and lethrinids, in which HPCs in well-sampled fishes were 19.43 and 22.25, respectively, and SLIPs were 10.57 and 9.50, respectively. Results for all fishes (including species with low sample size, but excluding anecdotal collections) constitute about half of these figures.

**Table 4:** Parasite biodiversity in reef-associated families: lutjanids and nemipterids compared with lethrinids and serranids, and a calculation on all four families

**Family or group**		**All data**	**Well-sampled**		**All data**	**Well- sampled**
**Lutjanidae, reef-associated**	**HPCs**	81	20	**HPCs mean**	**10.13**	**20.00**
(All data: 8 species*; well-sampled: 1 species**)	**SLIPs**	25	9	**SLIPs mean**	**3.13**	**9.00**
**Nemipteridae**	**HPCs**	42	25	**HPCs mean**	**14.00**	**25.00**
(All data: 3 species*; well-sampled: 1 species**)	**SLIPs**	15	13	**SLIPs mean**	**5.00**	**13.00**
**Lethrinidae**	**HPCs**	188	89	**HPCs mean**	**11.06**	**22.25**
(All data: 17 species; well-sampled: 4 species)	**SLIPs**	42	38	**SLIPs mean**	**2.47**	**9.50**
**Serranidae**	**HPCs**	337	136	**HPCs mean**	**12.04**	**19.43**
(All data: 28 species; well-sampled: 7 species)	**SLIPs**	75	74	**SLIPs mean**	**2.68**	**10.57**
**Four families of reef-associated fish**	**HPCs**	648	270	**HPCs mean**	**11.57**	**20.77**
(All data: 56 species; well-sampled: 13 species)***	**SLIPs**	148	125	**SLIPs mean**	**2.64**	**9.62**

*: species with only anecdotal data (see Table 1) were excluded from these calculations.

** species sampled over 30: Lutjanidae, only *Lutjanus vitta* (n = 42); Nemipteridae, only *Nemipterus furcosus* (n = 239).

*** SLIPs for all four families are not a simple addition of SLIPs for each families, because a few taxa are shared. These include only the digenean *Stephanostomum japonocasum* (shared by 2 fish families), and the four trypanorhynchs *C. gracilis* (shared by 4 families), *F. minacanthus*, *Ps. heroniensis* and *Ny. goreensis* (shared by 2–3 families).

In addition, Table 4 includes results obtained by pooling the data for all four families of fish. Results from 13 species of well-sampled reef-associated fishes (only n > 25 or n > 30 sampled individuals according to family), represented a sampling effort of almost 1,000 fishes (382 serranids, 329 lethrinids, 42 lutjanids, 239 nemipterids, total 992). The sampling effort and person-day time represented by these figures, which include collection, preparation, precise identification of parasite specimens, and curation in recognized collections, has no equivalent in the literature for reef-associated fish and all of their parasites (although similar efforts were devoted to digeneans only [4,116,117]). The number of HPCs is 20.77 per fish species, and the number of SLIPs is 9.62 per fish species. Many precautions were taken in these studies to *minimize* the number of taxa, and larval taxa (which are difficult to identify) certainly represent a significant additional biodiversity. We thus consider that the total result of *ten species of parasites per reef fish species* is a strict minimum, and that the real number is probably double or triple this estimate. All numbers here concern only macroparasites; the addition of Myxosporea, if they were known, would significantly enhance all results, with probably 2–3 additional parasite species per fish species [118,119].

Our published [6,81,120-124] and unpublished data on other families of fish suggest that this number of 10 species of parasites per fish species is generalizable to other families of fish, at least those with similar “average” size (10–40 cm), since parasite biodiversity depends upon the size of fish [4,8].

As found for serranids and lethrinids [7,8], the literature includes very few extensive lists of parasites from lutjanids and nemipterids in the Pacific. The checklist of parasites of Heron Island [44] includes a single lutjanid species in common with the present study, *Lu. fulviflamma*, with a single parasite record, and no nemipterid species in common. This precludes any biogeographical comparison for lutjanids and nemipterids, as was found for serranids and lethrinids [7,8].

### Consequences for coextinction of parasites of coral reef fish

The word coextinction was coined by Stork and Lyal (1993) [125] to express that as a host species becomes extinct, so does one or more species of parasites, and was redefined by Koh et al. (2004) [126] in a slightly broader sense as “the loss of a species (the affiliate) upon the loss of another (the host)”.

Knowing that parasite species are more numerous than non-parasitic species [127,128], it follows that coextinctions are more numerous than extinctions [129]. Dobson et al. (2008) [130] estimated that 3–5% of helminths are threatened by extinction in the next 50–100 years. However, Dunn (2009) [129] mentioned that there is no well documented case of the coextinction of a vertebrate parasite. Rózsa (1992) [131] pointed out that even a decrease in numbers within a host population, without the danger of extinction, could jeopardize the survival of certain parasite species. Koh et al. (2004) [126] calculated a risk of extinction of 593 species of monogeneans associated with 746 endangered species of fish. Justine (2007) [132] remarked that such a prediction underestimated the number of parasites in fish in rich ecosystems such as coral reefs. For Moir et al (2010) [133], this discrepancy highlights how biogeographic variation and knowledge gaps in dependant species biodiversity may lead to biased estimates of coextinction risk.

Should we be concerned by the extinction of parasites? This is hard to defend to the general public, because “parasites tend to lack charisma” [129] and many blood-sucking parasites transmit diseases [134]. However, parasites play a major role in the balance of populations and the evolution of their hosts [114,129,130,135], and, as such, are an important and irreplaceable part of biodiversity and ecosystems.

The numerical evaluation of parasitic biodiversity in coral reef fish provided in the present study allows a more precise prediction of the risk of coextinction if a coral reef fish species becomes extinct, or simply has its population decreased [133]. Coral reefs are threatened across the planet [136-140] and special threats exist in New Caledonia [141,142]. Our results suggest that extinction of a coral reef fish species of average size would eventually result in the co-extinction of at least ten species of parasites.

## Conclusions

As surprising as it might seem for studies mainly based on lists of parasites and morphological identifications, the present paper and our two previous similar papers [7,8] are pioneering works in the field of biodiversity of parasites of tropical coral reef fishes. Our main discovery of a parasitic biodiversity at least ten times higher than fish biodiversity has potential implications in the evaluation of loss of biodiversity when a coral reef fish species is threatened or becomes extinct.

## Methods

Methods used in this paper are essentially the same as for the two previous papers of this series [7,8] and for brevity are not repeated here. For parasite collection, we used two methods targeting two sets of organs, designated as “gills” and “abdominal organs”. We generally used the “gut wash” method [143] but in some circumstances we used a simplified method [144]. Full details and possible methodological flaws were discussed previously [7,8]. In addition, we soaked a few fish in saline in order to collect surface monogeneans. A high number (239) of fork-tailed threadfin breams, *Nemipterus furcosus*, were examined, including specimens examined for research and specimens examined by students during practical courses at the University of New Caledonia.

Parasite specimens, generally collected by J.-L. Justine and his team, and sometimes by visiting colleagues, were forwarded to respective specialists: I. Beveridge (trypanorhynch cestodes), G. A. Boxshall (copepods), R. A. Bray and T. L. Miller (digeneans), F. Moravec (nematodes), J.-P. Trilles (isopods), I. D. Whittington (capsalid monogeneans), and J.-L. Justine (other monogeneans). Hirudineans were examined by E. Burreson (College of William and Mary, Gloucester Point, Virginia, USA); a few monogeneans were examined by L. Euzet (Sète, France); some anisakids were identified at the generic level by S. Shamsi (Charles Sturt University, Wagga Wagga, NSW, Australia). Gills of several lutjanids, prepared with hot water and formalin, were examined by D. C. Kritsky (Idaho State University, Pocatello, Idaho, USA) and monogeneans were described [45]. Sometimes external isopods were brought by fishermen and provided “anecdotal” collections.

The names of cestode orders and suborders follow Khalil et al. [145] updated by recent work [98,146,147]. Polyopisthocotylean and monopisthocotylean monogeneans are treated as two independent groups, because monophyly of the monogeneans is not established [148-152]. However, since polyopisthocotyleans were rare, results for both groups were often pooled. Monogeneans sometimes included in the Dactylogyridae are here considered as members of the Ancyrocephalidae [153,154]. Many specimens have been deposited in recognized collections (Appendix 3); other specimens under study are still in the collections of the various authors but will be eventually deposited in the collection of the Muséum National d'Histoire Naturelle, Paris, France (MNHN) and/or in other recognized, curated collections.

Research carried out on animals (fish) was performed in accordance with the ethical requirements of the IRD (Institut de Recherche pour le Développement, France) and University of Adelaide Animal Ethics Approval S-020-2008 for work by I.D. Whittington.

## Appendix 1. Host-parasite list

New, unpublished records indicated by [0]; other records: [6,24,25,45,47,58,59,71,78,79,81,87,88,107,155-166].

8 fish species with low sample size * were included in Table 1 but not kept in final calculations of parasite numbers (Table 3).

### Caesionidae

#### *Caesio cuning* (Bloch, 1791)

**Dige:** Hemi: *Lecithochirium* sp. (digestive tract) [0]

**Dige:** Leci: *Aponurus* sp. (digestive tract) [0]

**Both:** Unid: unidentified larvae (digestive tract) [0]

**Tetr:** Unid: unidentified larvae (digestive tract) [0]

**Nema:** Cucu: *Cucullanus* sp. (digestive tract) [0]

Remarks: 8 specimens examined (4 for gills, 8 for abdominal organs)HPCs: 5; SLIP-HPCs: 0.

#### Lutjanidae, reef-associated

##### *Aprion virescens* Valenciennes, 1830

**Mono:** Caps: *Pseudonitzschia uku* (gills) [58]

**Dige:** Acan: *Pleorchis uku*, immature (intestine) [81]

**Dige:** Acan: *Stephanostomum uku* (intestine) [81]

Remarks: 3 specimens examined (3 for gills, 3 for abdominal organs)HPCs: 3; SLIP-HPCs: 3.

##### *Lutjanus adetii* (Castelnau, 1873)

**Dige:** Opec: *Hamacreadium mutabile* (digestive tract) [0]

**Dige:** Scle: *Prosogonotrema bilabiatum* immature (digestive tract) [0] (NGR)

**Both:** Unid: unidentified larvae (digestive tract) [0]

**Nema:** Anis: unidentified larvae (abdominal cavity) [0]

Remarks: 5 specimens examined (0 for gills, 5 for abdominal organs)HPCs: 4; SLIP-HPCs: 2.

##### *Lutjanus argentimaculatus* (Forsskål, 1775)

**Isop:** Gnat: Praniza larvae (gills) [0]

**Cope:** Hats: *Hatschekia* n. sp. 20 (gills) [0]

**Cope:** Lerp: *Parabrachiella lutiani* (gills) [0] (NHR)

**Mono:** Ancy: *Euryhaliotrema* sp. (gills) [45]

**Mono:** Ancy: *Haliotrematoides novaecaledoniae* (gills) [45]

**Mono:** Ancy: *Haliotrematoides potens* (gills) [45]

**Mono:** Caps: *Metabenedeniella* sp. (pectoral fins) [59]

**Mono:** Caps: *Trilobiodiscus lutiani* (gills) [59] (NGR)

**Dige:** Cryp: *Retrovarium manteri* (digestive tract) [0] (NGR)

**Dige:** Cryp: *Retrovarium saccatum* (digestive tract) [0] (NHR)

**Dige:** Cryp: *Siphoderina hirastricta* (digestive tract) [0] (NGR)

**Dige:** Opec: *Hamacreadium mutabile* (digestive tract) [0] (NHR)

**Tetr:** Unid: unidentified larvae (digestive tract) [0]

Remarks: 4 specimens examined (3 for gills, 3 for abdominal organs)HPCs: 13; SLIP-HPCs: 8.

##### *Lutjanus fulviflamma* (Forsskål, 1775)

**Cope:** Cali: Chalimus larvae (gills) [0]

**Cope:** Hats: *Hatschekia tanysoma* (gills) [0] (NGR)

**Mono:** Ancy: *Euryhaliotrema* sp.(gills) [45]

**Mono:** Ancy: *Haliotrematoides patellacirrus* (gills) [45]

**Mono:** Ancy: *Haliotrematoides tainophallus* (gills) [45]

**Poly:** Unid (gills) [0]

**Dige:** Cryp: *Euryakaina marina* (intestine) [78]

**Dige:** Opec: *Hamacreadium mutabile* (digestive tract) [0]

**Nema:** Anis: unidentified larvae (abdominal cavity) [0]

Remarks: 17 specimens examined (11 for gills, 11 for abdominal organs)HPCs: 9; SLIP-HPCs: 5.

##### *Lutjanus fulvus* (Forster, 1801) *

**Mono:** Ancy: *Euryhaliotrema* sp. (gills) [45]

**Mono:** Ancy: *Haliotrematoides longitubocirrus* (gills) [45]

**Mono:** Ancy: *Haliotrematoides patellacirrus* (gills) [45]

**Mono:** Ancy: unidentified (gills) [45]

Remarks: 2 specimens examined (1 for gills, 0 for abdominal organs, additional gills examined by D. C. Kritsky). * Fish species not kept for final parasite counts (Table 3) because of low sample size.HPCs: 4; SLIP-HPCs: 2.

##### *Lutjanus gibbus* (Forsskål, 1775) *

**Cope:** Hats *Hatschekia clava* (gills) [0] (NHR)

**Mono:** Ancy: unidentified (gills) [0]

Remarks: 2 specimens examined (2 for gills, 1 for abdominal organs). * Fish species not kept for final parasite counts (Table 3) because of low sample size.HPCs: 2; SLIP-HPCs: 1.

##### *Lutjanus kasmira* (Forsskål, 1775)

**Cope:** Hats: *Hatschekia* n. sp. 19 (gills) [0]

**Mono:** Dipl: *Diplectanum* cf. *fusiforme* (gills) [0]

**Mono:** Dipl: *Diplectanum* cf. *spirale* (gills) [0]

**Mono:** Ancy: unidentified sp. 1 (gills) [0] [45]

**Mono:** Ancy: unidentified sp. 2 (gills) [0]

**Mono:** Ancy: unidentified sp. 3 (gills) [0]

**Mono:** Ancy: unidentified sp. 4 (gills) [0]

**Dige:** Cryp: *Siphoderina* sp. (digestive tract) [0]

**Dige:** Didy: unidentified larvae (digestive tract) [0]

**Dige:** Hemi: *Lecithochirium* sp. (digestive tract) [0]

**Dige:** Opec: *Hamacreadium mutabile* (digestive tract) [163]

**Dige:** Tran: *Transversotrema borboleta* (under scales) [0] (NGR)

**Both:** Unid: unidentified larvae (digestive tract) [0]

**Tetr:** Unid: unidentified larvae (digestive tract) [0]

**Nema:** Anis: unidentified larvae (abdominal cavity) [0]

Remarks: 16 specimens examined (12 for gills, 12 for abdominal organs, 2 soaked bodies for skin parasites, additional gills examined by D. C. Kritsky)HPCs: 15; SLIP-HPCs: 1.

##### *Lutjanus monostigma* (Cuvier, 1828) *

**Isop:** Cora: *Argathona macronema* (body) [0] (NHR)

Remarks: 0 specimen examined (external isopod collected from one fish otherwise not examined). * Fish species not kept for final parasite counts (Table 3) because of low sample size.HPCs: 1; SLIP-HPCs: 1.

##### *Lutjanus quinquelineatus* (Bloch, 1790)

**Mono:** Ancy: *Euryhaliotrema* sp. (gills) [45]

**Mono:** Ancy: *Haliotrematoides lanx* (gills) [45]

**Mono:** Ancy: *Haliotrematoides longitubocirrus* (gills) [45]

**Mono:** Ancy: *Haliotrematoides patellacirrus* (gills) [45]

**Mono:** Ancy: unidentified (gills) [45]

**Dige:** Cryp: *Varialvus charadrus* (intestine) [79]

**Dige:** Opec: *Hamacreadium mutabile* (intestine) [0]

**Tetr:** Unid: unidentified larvae (digestive tract) [0]

**Nema:** Anis: unidentified larvae (abdominal cavity) [0]

Remarks: 12 specimens examined (0 for gills, 6 for abdominal organs, additional gills examined by D. C. Kritsky)HPCs: 9; SLIP-HPCs: 5.

##### *Lutjanus rivulatus* (Cuvier, 1828) *

**Mono:** Ancy: unidentified sp. (gills) [0]

**Nema:** Anis: unidentified larvae (abdominal cavity) [0]

**Hiru:** Pisc: *Trachelobdella* sp. immature (gills) [0]

Remarks: 2 specimens examined (2 for gills, 2 for abdominal organs; including 1 juvenile). * Fish species not kept for final parasite counts (Table 3) because of low sample size.HPCs: 3; SLIP-HPCs: 0.

##### *Lutjanus russellii* (Bleeker, 1849)

**Isop:** Gnat: Praniza larvae (gills) [0]

**Mono:** Ancy: *Euryhaliotrema* sp. (gills) [45]

**Mono:** Ancy: *Haliotrematoides longitubocirrus* (gills) [45]

**Mono:** Ancy: *Haliotrematoides patellacirrus* (gills) [45]

**Mono:** Caps: Capsalidae sp. 6 (body washing) [59]

**Poly:** Unid: unidentified immature (gills) [0]

**Dige:** Didy: unidentified larvae (intestine) [0]

**Dige:** Opec: *Hamacreadium mutabile* (intestine) [0]

Remarks: 6 specimens examined (0 for gills, 1 for abdominal organs, additional gills examined by D. C. Kritsky)HPCs: 8; SLIP-HPCs: 3.

##### *Lutjanus vitta* (Quoy & Gaimard, 1824)

**Cope:** Cali: chalimus larvae (gills) [0]

**Mono:** Ancy: *Euryhaliotrema* sp. (gills) [45]

**Mono:** Ancy: *Haliotrematoides longitubocirrus* (gills) [45]

**Mono:** Ancy: *Haliotrematoides patellacirrus* (gills) [45]

**Mono:** Ancy: unidentified (gills) [45]

**Mono:** Caps: Capsalidae sp. 7 (branchiostegal membranes) [59]

**Mono:** Dipl: *Diplectanum* cf. *fusiforme* (gills) [0]

**Dige:** Cryp: *Euryakaina manilensis* (intestine) [78]

**Dige:** Cryp: *Varialvus charadrus* (intestine) [79]

**Dige:** Didy: unidentified larvae (intestine) [0]

**Dige:** Hemi: *Lecithochirium* sp. (intestine) [0]

**Dige:** Opec: *Hamacreadium mutabile* (intestine) [0] (NHR)

**Tetr:** Unid: unidentified (intestine) [0]

**Tryp:** Laci: *Callitetrarhynchus gracilis* (abdominal cavity) [0]

**Tryp:** Laci: *Pseudolacistorhynchus heroniensis* (abdominal cavity) [0] (NHR)

**Nema:** Anis: *Raphidascaris (Ichthyascaris)* sp. (intestine) [161]

**Nema:** Anis: *Terranova* sp. larvae (abdominal cavity) [0]

**Nema:** Cama: unidentified larvae (intestine) [0]

**Nema:** Phil: *Philometra brevicollis* (gonads) [160]; as “sp” [165]

**Nema:** Phil: *Philometra mira* (gonads) [160]; as “sp” [165]

Remarks: 42 specimens examined (19 for gills, 31 for abdominal organs, 5 unproductive soaked bodies, additional gills examined by D. C. Kritsky)HPCs: 20; SLIP-HPCs: 9.

##### *Macolor niger* (Forsskål, 1775) *

**Cope:** Cali: *Caligus* n. sp. 2 (gills) [0]

**Cope:** Diss: *Dissonus excavatus* (gills) [155]

**Cope:** Hats: *Hatschekia* n. sp. 18 (gills) [0]

**Mono:** Caps: unidentified (gills) [0]

**Mono:** Gyro: unidentified (gills) [0]

**Dige:** Hemi: *Lecithochirium* sp. (intestine) [0]

Remarks: 2 specimens examined (2 for gills, 1 for abdominal organs). * Fish species not kept for final parasite counts (Table 3) because of low sample size.HPCs: 6; SLIP-HPCs: 1.

#### Lutjanidae, deep water

##### *Etelis carbunculus* Cuvier, 1828

**Isop:** Cymo: *Anilocra gigantea* (body) [0]

**Cope:** Cali: *Caligus brevis* (body) [0] (NHR)

**Cope:** Cali: chalimus larvae (gills) [0]

**Cope:** Hats: *Hatschekia* n. sp. 21 (gills) [0]

**Cope:** Lerp: *Parabrachiella* sp. 2 (body) [0]

**Mono:** Caps: *Benedenia elongata* (gills) [0] (NHR)(NGR)

**Dige:** Cryp: *Siphoderina ulaula* (digestive tract) [0] (NGR)

**Dige:** Didy: unidentified adults (under scales) [0]

**Dige:** Opec: *Neolebouria blatta* (digestive tract) [87]

**Tetr:** Unid: unidentified larvae (digestive tract) [0]

**Tryp:** Unid: unidentified larvae, sterile cysts (abdominal cavity) [0]

**Nema:** Anis: *Raphidascaris (Ichthyascaris)* sp. (digestive tract) [0]

**Nema:** Cucu: *Dichelyne etelidis* (digestive tract) [159]

**Acan:** Unid: unidentified (digestive tract) [0]

Remarks: 16 specimens examined (5 for gills, 3 for abdominal organs, occasional collect of skin isopods or didymozoids)HPCs: 14; SLIP-HPCs: 6.

##### *Etelis coruscans* Valenciennes, 1862

**Isop:** Cymo: *Anilocra gigantea* (body) [0] (NHR)

**Cope:** Cali: *Caligus brevis* (body) [0] (NHR)

**Cope:** Cali: chalimus larvae (body) [0]

**Cope:** Penn: chalimus larvae and premetamorphic adults (body) [0]

**Cope:** Hats: *Hatschekia* n. sp. 21 (gills) [0]

**Mono:** Caps: *Benedenia elongata* (gills) [0] (NHR)(NGR)

**Mono:** Caps: *Lagenivaginopseudobenedenia etelis* (gills) [0] (NHR)(NGR)

**Poly:** Micr: *Allomicrocotyla* sp. (gills) Euzet det.

**Dige:** Cryp: *Siphoderina* cf. *onaga* (digestive tract) [0]

**Dige:** Cryp: *Siphoderina* n. sp. (digestive tract) [0]

**Dige:** Fell: *Tergestia magna* (digestive tract) [0] (NGR)(NHR)

**Tryp:** Unid: unidentified larvae, sterile cysts (abdominal cavity) [0]

**Nema:** Anis: *Raphidascaris (Ichthyascaris) etelidis* (digestive tract) [161]

**Nema:** Cucu: *Dichelyne etelidis* (digestive tract) [159]

Remarks: 18 specimens examined (11 for gills, 5 for abdominal organs, occasional collect of skin isopods or didymozoids); The taxon listed as *Lagenivaginopseudobenedenia* sp. and sequenced in [59] is actually likely to be *Benedenia elongata* (I.D. Whittington, unpublished)HPCs: 14; SLIP-HPCs: 7.

##### *Pristipomoides argyrogrammicus* (Valenciennes, 1832)

**Isop:** Cymo: *Anilocra longicauda* (body) [0] (NHR)(NGR)

**Cope:** Lerp: *Clavellotis* sp. (pectoral fins) [6,162]

**Mono:** Caps: *Benedenia elongata* (gills) [0] (NHR)(NGR)

**Mono:** Caps: Capsalidae sp. 17 (head) [59]

**Mono:** Dipl: *Diplectanum* cf. *curvivagina* (gills) [0]

**Dige:** Cryp: *Metadena rooseveltiae* (digestive tract) [0] (NHR)(NGR)

**Dige:** Cryp: *Siphoderina* n. sp. (digestive tract) [0]

**Dige:** Cryp: *Siphoderina ulaula* (digestive tract) [0] (NHR)(NGR)

**Dige:** Opec: *Neolebouria blatta* (digestive tract) [87]

**Tetr:** Unid: unidentified (intestine) [0]

**Tryp:** Unid: unidentified larvae, sterile cysts (abdominal cavity) [0]

**Nema:** Anis: unidentified larvae (abdominal cavity) [0]

**Nema:** Capi: *Pseudocapillaria novaecaledoniensis* (digestive tract) [158]

Remarks: 20 specimens examined (14 for gills, 18 for abdominal organs, 1 soaked body). The taxon listed as Capsalidae sp. 17 and sequenced in [166] (no mounted voucher specimen available) is actually likely to be *Benedenia elongata* (I.D. Whittington, 

##### *Pristipomoides auricilla* (Jordan, Evermann & Tanaka, 1927)

**Isop:** Gnat: Praniza larvae (gills) [0]

**Mono:** Dipl: *Diplectanum* cf. *opakapaka* (gills) [0]

**Dige:** Lepo: *Lepidapedoides kalikali* (stomach) [0] (NGR)

**Tetr:** Unid: unidentified larvae (intestine) [0]

**Tryp:** Unid: unidentified larvae, sterile cysts (abdominal cavity) [0]

**Nema:** Anis: unidentified larvae (abdominal cavity) [0]

**Nema:** Cama: unidentified adults (digestive tract) [0]

**Nema:** Cucu: *Cucullanus bourdini* (digestive tract) [159]

Remarks: 2 specimens examined (2 for gills, 2 for abdominal organs)HPCs: 8; SLIP-HPCs: 2.

##### *Pristipomoides filamentosus* (Valenciennes, 1830) *

**Isop:** Aegi: *Aega musorstom* (body) [0] (NHR)

**Isop:** Cymo: *Anilocra gigantea* (body) [0] (NHR)

**Cope:** Penn: *Lernaeolophus sultanus* (body) [0] (NHR)

**Mono:** Dipl: *Diplectanum* sp. (gills) [0]

**Dige:** Didy: unidentified adults (digestive tract) [0]

**Dige:** Didy: unidentified larvae (digestive tract) [0]

**Nema:** Anis: *Raphidascaris (Ichthyascaris) etelidis* (digestive tract) [161]

**Nema:** Cama: *Camallanus* sp. (digestive tract) [0]

**Nema:** Cucu: *Cucullanus bourdini* (digestive tract) [159]

Remarks: 7 specimens examined (2 for gills, 2 for abdominal organs, occasional collect of external isopods or copepods). * Fish species not kept for final parasite counts (Table 3) because of low sample size.HPCs: 9; SLIP-HPCs: 5.

#### Nemipteridae

##### *Nemipterus furcosus* (Valenciennes, 1830)

**Isop:** Gnat: Praniza larvae (gills) [0]

**Mono:** Caps: unidentified (gills) (“*Benedenia* sp.” [156])

**Mono:** Caps: Capsalidae sp. 13 (pelvic and anal fins, gills, branchiostegal membranes) [0] (branchiostegal membranes) [59]

**Poly:** Micr: unidentified immature (gills) [156]

**Dige:** Cryp: *Adlardia novaecaledoniae* (intestine) [71,164]

**Dige:** Didy: unidentified larvae (digestive tract) [0]

**Dige:** Hemi: *Ectenurus trachuri* (digestive tract) [0] (NGR)(NHR)

**Dige:** Opec: *Macvicaria jagannathi* (digestive tract) [87]

**Dige:** Opec: *Neolebouria lineatus* (digestive tract) [87]

**Both:** Unid: unidentified larvae (abdominal cavity and intestine) [0]

**Tetr:** Unid: unidentified larvae (intestine) [0]

**Tryp:** Laci: *Callitetrarhynchus gracilis* larvae (abdominal cavity) [0] (NHR)

**Tryp:** Laci: *Floriceps minacanthus* larvae (abdominal cavity) [0]

**Tryp:** Otob: *Otobothrium mugilis* larvae (intestine) [0]

**Tryp:** Tent: *Nybelinia goreensis* larvae (intestine) [0]

**Tryp:** Tent: *Nybelinia indica* larvae (intestine) [0] (NHR)(NGR)

**Tryp:** Tent: *Nybelinia queenslandensis* larvae (intestine) [0] (NHR)(NGR)

**Nema:** Anis: *Anisakis* sp. larvae (abdominal cavity) [0]

**Nema:** Anis: *Hysterothylacium* sp. larvae (abdominal cavity) [0]

**Nema:** Anis: *Raphidascaris (Ichthyascaris) nemipteri* adults (intestine) [157]

**Nema:** Cama: *Camallanus carangis* adults (intestine) [166]

**Nema:** Gnto: unidentified larvae (intestine) [0]

**Nema:** Phys: unidentified larvae (intestine) [0]

**Nema:** Tric: *Huffmanela branchialis* eggs (gills) [24]

**Turb:** Unid: unidentified adults (gills) [107]

Remarks: 239 specimens examined (111 for gills, 160 for abdominal organs, branchiostegal membranes examined)HPCs: 25; SLIP-HPCs: 13.

##### *Pentapodus aureofasciatus* Russell, 2001

**Isop:** Gnat: Praniza larvae (gills) [156]

**Cope:** Hats: *Hatschekia* n. sp. 17 (gills) [0]; as “sp.” [47]

**Mono:** Ancy: unidentified (gills) [0]

**Mono:** Dipl: *Calydiscoides limae* (gills) [156]

**Poly:** Micr: unidentified immature (gills) [156]

**Dige:** Hemi: *Lecithochirium* sp. (digestive tract) [0]

**Dige:** Hemi: *Lecithocladium* sp. (digestive tract) [0]

**Dige:** Opec: *Neochoanostoma* sp. (digestive tract) [0]

**Dige:** Opec: *Neolebouria* sp. (digestive tract) [0]

**Tetr:** Unid: unidentified larvae (intestine) [0]

**Nema:** Anis: unidentified larvae (abdominal cavity) [0]

**Nema:** Tric: *Huffmanela* sp. eggs (gills) [24][25]

Remarks: 23 specimens examined (19 for gills, 12 for abdominal organs)HPCs: 12; SLIP-HPCs: 1.

##### *Pentapodus nagasakiensis* (Tanaka, 1915) *

**Dige:** Hemi: *Lecithochirium* sp. (digestive tract) [0]

**Dige:** Opec: *Macvicaria* sp. (digestive tract) [0]

**Tetr:** Unid: unidentified larvae (intestine) [0]

**Nema:** Anis: unidentified larvae (abdominal cavity) [0]

Remarks: 2 specimens examined (2 for gills, 2 for abdominal organs). * Fish species not kept for final parasite counts (Table 3) because of low sample size.HPCs: 4; SLIP-HPCs: 0.

##### *Scolopsis bilineata* (Bloch, 1793)

**Isop:** Gnat: Praniza larvae (gills) [0]

**Dige:** Monr: *Allobacciger macrorchis* (intestine) [0] (NGR)(NHR)

**Dige:** Opec: *Allopodocotyle* sp. (digestive tract) [0]

**Nema:** Cama: unidentified larvae (intestine) [0]

**Hiru:** Pisc: *Trachelobdella* sp. immature (gills) [0]

Remarks: 12 specimens examined (8 for gills, 9 for abdominal organs)HPCs: 5; SLIP-HPCs: 1.

##### *Scolopsis taenioptera* (Cuvier, 1830) *

**Nema:** Anis: unidentified larvae (abdominal cavity) [0]

Remarks: 3 specimens examined (2 for gills, 3 for abdominal organs). * Fish species not kept for final parasite counts (Table 3) because of low sample size.HPCs: 1; SLIP-HPCs: 0.

### Appendix 2. Parasite-host list

8 fish species with low sample size * were included in Table 1 but not kept in final calculations of parasite numbers (Table 3).

#### Isopoda

Minimized number of taxa: 5

Number of SLIPs: total 4; Lutjanidae: reef: 1-0*, deep-sea: 3-3*, all: 4-3*; Nemipteridae: 0.

Number of non-SLIP taxa: 0

Undistinguishable larval taxa: 1 (gnathiids)

**Aegi:***Aega musorstom*

*Pristipomoides filamentosus* (NHR)

**Cora:***Argathona macronema*

Lutjanus monostigma * (NHR)

**Cymo:***Anilocra gigantea*

Etelis carbunculus

*Etelis coruscans* (NHR)

*Pristipomoides filamentosus* (NHR)

**Cymo:***Anilocra longicauda*

*Pristipomoides argyrogrammicus* (NHR)(NGR)

**Gnat:** Praniza larvae

Lutjanus argentimaculatus

Lutjanus russellii

Nemipterus furcosus

Pentapodus aureofasciatus

Pristipomoides auricilla *

Scolopsis bilineata

#### Copepoda

Minimized number of taxa: 14

Number of SLIPs: total 6; Lutjanidae: reef: 4-2*; deep-sea: 2-2*, all: 6-4*; Nemipteridae: 0-0*.

Undistinguishable larval taxa: 2

Note: for minimizing number of taxa, we considered that the caligid and pennellid larvae could correspond to their adult counterparts found on same or similar fish.

**Cali:***Caligus brevis*

*Etelis carbunculus* (NHR)

*Etelis coruscans* (NHR)

**Cali:***Caligus* n. sp. 2

Macolor niger *

**Cali:** chalimus larvae

Etelis carbunculus

Etelis coruscans

Lutjanus fulviflamma

Lutjanus vitta

**Diss:***Dissonus excavatus*

Macolor niger *

**Hats:***Hatschekia clava*

Lutjanus gibbus * (NHR)

**Hats:***Hatschekia* n. sp. 17

Pentapodus aureofasciatus

**Hats:***Hatschekia* n. sp. 18

Macolor niger *

**Hats:***Hatschekia* n. sp. 19

Lutjanus kasmira

**Hats:***Hatschekia* n. sp. 20

Lutjanus argentimaculatus

**Hats:***Hatschekia* n. sp. 21

Etelis carbunculus

Etelis coruscans

**Hats:***Hatschekia tanysoma*

*Lutjanus fulviflamma* (NGR)

**Lerp:***Clavellotis* sp.

Pristipomoides argyrogrammicus

**Lerp:***Parabrachiella lutiani*

*Lutjanus argentimaculatus* (NHR)

**Lerp:***Parabrachiella* sp. 2

Etelis carbunculus

**Penn:** chalimus larvae and premetamorphic adults

Etelis coruscans

**Penn:***Lernaeolophus sultanus*

*Pristipomoides filamentosus* (NHR)

#### Monopisthocotylea

Minimized number of taxa: 23

Number of SLIPs: total 11; Lutjanidae: reef: 8-8*, deep-sea: 2-2*, all: 10-10*; Nemipteridae: 1-1*.

Number of non-SLIP taxa: 10

Undistinguishable larval taxa: 0

Note: Note: for minimizing number of taxa, *Euryhaliotrema* spp. and Ancyrocephalids Gen. spp. were each counted as one species (but this is certainly an underestimate), and the two unidentified capsalids and *Diplectanum* sp. were not counted (because they could, respectively, correspond to their better identified counterparts).

**Ancy:***Euryhaliotrema* sp. (several spp.)

Lutjanus argentimaculatus

Lutjanus fulviflamma

Lutjanus fulvus *

Lutjanus quinquelineatus

Lutjanus russellii

Lutjanus vitta

**Ancy:***Haliotrematoides lanx*

Lutjanus quinquelineatus

**Ancy:***Haliotrematoides longitubocirrus*

Lutjanus fulvus *

Lutjanus quinquelineatus

Lutjanus russellii

Lutjanus vitta

**Ancy:***Haliotrematoides novaecaledoniae*

Lutjanus argentimaculatus

**Ancy:***Haliotrematoides patellacirrus*

Lutjanus fulviflamma

Lutjanus fulvus *

Lutjanus quinquelineatus

Lutjanus russellii

Lutjanus vitta

**Ancy:***Haliotrematoides potens*

Lutjanus argentimaculatus

**Ancy:***Haliotrematoides tainophallus*

Lutjanus fulviflamma

**Ancy:** unidentified sp. (often several sp.)

Lutjanus fulvus *

Lutjanus gibbus *

Lutjanus kasmira

Lutjanus quinquelineatus

Lutjanus rivulatus *

Lutjanus vitta

Pentapodus aureofasciatus

**Caps:***Benedenia elongata*

*Etelis carbunculus* (NHR)(NGR)

*Etelis coruscans* (NHR)(NGR)

*Pristipomoides argyrogrammicus* (NHR)(NGR)

**Caps:** Capsalidae sp. 6

Lutjanus russellii

**Caps:** Capsalidae sp. 7

Lutjanus vitta

**Caps:** Capsalidae sp. 13

Nemipterus furcosus

**Caps:** Capsalidae sp. 17

Pristipomoides argyrogrammicus

**Caps:***Lagenivaginopseudobenedenia etelis*

*Etelis coruscans* (NHR)(NGR)

**Caps:***Metabenedeniella* sp.

Lutjanus argentimaculatus

**Caps:***Pseudonitzschia uku*

Aprion virescens

**Caps:***Trilobiodiscus lutiani*

*Lutjanus argentimaculatus* (NGR)

**Caps:** unidentified species

Macolor niger *

Nemipterus furcosus

**Dipl:***Calydiscoides limae*

Pentapodus aureofasciatus

**Dipl:***Diplectanum* cf. *curvivagina*

Pristipomoides argyrogrammicus

**Dipl:***Diplectanum* cf. *fusiforme*

Lutjanus kasmira

Lutjanus vitta

**Dipl:***Diplectanum* cf. *opakapaka*

Pristipomoides auricilla *

**Dipl:***Diplectanum* cf. *spirale*

Lutjanus kasmira

**Dipl:***Diplectanum* sp.

Pristipomoides filamentosus

**Gyro:** unidentified species

Macolor niger *

#### Polyopisthocotylea

Minimized number of taxa: 2

Number of SLIPs: 0

Number of non-SLIP taxa: 1

Undistinguishable larval taxa: 0

Note: for minimizing number of taxa, we considered all unidentified records as a single species.

**Micr:***Allomicrocotyla* sp.

Etelis coruscans

**Micr:** unidentified species

Nemipterus furcosus

Pentapodus aureofasciatus

unidentified family: unidentified species

Lutjanus fulviflamma

Lutjanus russellii

#### Digenea

Minimized number of taxa: 33

Number of SLIPs: total 20; Lutjanidae: reef: 10-10*, deep-sea: 5-4*, all 15-14*; Nemipteridae: 5-5*.

Number of non-SLIP taxa: 2

Undistinguishable larval taxa: 1 (didymozoid juveniles)

Note: for minimizing number of taxa, we considered that the single non-SLIP taxon was *Siphoderina* cf. *onaga*; the 2 *Siphoderina* sp. from deep-sea and coral lutjanids were counted as 2 taxa; unidentified adult didymozoids and unidentified juveniles were counted each as 1 taxon.

**Acan:***Pleorchis uku*

Aprion virescens

**Acan:***Stephanostomum uku*

Aprion virescens

**Cryp:***Adlardia novaecaledoniae*

Nemipterus furcosus

**Cryp:***Euryakaina manilensis*

Lutjanus vitta

**Cryp:***Euryakaina marina*

Lutjanus fulviflamma

**Cryp:***Metadena rooseveltiae*

*Pristipomoides argyrogrammicus* (NHR)(NGR)

**Cryp:***Retrovarium manteri*

*Lutjanus argentimaculatus* (NGR)

**Cryp:***Retrovarium saccatum*

*Lutjanus argentimaculatus* (NHR)

**Cryp:***Siphoderina* cf. *onaga*

Etelis coruscans

**Cryp:***Siphoderina hirastricta*

Lutjanus argentimaculatus

**Cryp:***Siphoderina* n. sp.

Etelis coruscans

Pristipomoides argyrogrammicus

**Cryp:***Siphoderina* sp.

Lutjanus kasmira

**Cryp:***Siphoderina ulaula*

*Etelis carbunculus*(NGR)

*Pristipomoides argyrogrammicus* (NHR)(NGR)

**Cryp:***Varialvus charadrus*

Lutjanus quinquelineatus

Lutjanus vitta

**Didy:** unidentified adults

Etelis carbunculus

Pristipomoides filamentosus

**Didy:** unidentified juveniles

Lutjanus kasmira

Lutjanus russellii

Lutjanus vitta

Nemipterus furcosus

Pristipomoides filamentosus

**Fell:***Tergestia magna*

*Etelis coruscans* (NHR)(NGR)

**Hemi:***Ectenurus trachuri*

*Nemipterus furcosus* (NHR)(NGR)

**Hemi:***Lecithochirium* sp.

Caesio cuning

Lutjanus kasmira

Lutjanus vitta

Macolor niger *

Pentapodus nagasakiensis *

Pentapodus aureofasciatus

**Hemi:***Lecithocladium* sp.

Pentapodus aureofasciatus

**Leci:***Aponurus* sp.

Caesio cuning

**Lepo:***Lepidapedoides kalikali*

Pristipomoides auricilla * (NGR)

**Monr:***Allobacciger macrorchis*

*Scolopsis bilineata* (NHR)(NGR)

**Opec:***Allopodocotyle* sp.

Scolopsis bilineata

**Opec:***Hamacreadium mutabile*

Lutjanus adetii

*Lutjanus argentimaculatus* (NHR)

Lutjanus fulviflamma

Lutjanus kasmira

Lutjanus quinquelineatus

Lutjanus russellii

*Lutjanus vitta* (NHR)

**Opec:***Macvicaria jagannathi*

Nemipterus furcosus

**Opec:***Macvicaria* sp.

Pentapodus nagasakiensis *

**Opec:***Neochoanostoma* sp.

Pentapodus aureofasciatus

**Opec:***Neolebouria blatta*

Etelis carbunculus

Pristipomoides argyrogrammicus

**Opec:***Neolebouria lineatus*

Nemipterus furcosus

**Opec:***Neolebouria* sp.

Pentapodus aureofasciatus

**Scle:***Prosogonotrema bilabiatum*

*Lutjanus adetii* (NGR)

**Tran:***Transversotrema borboleta*

*Lutjanus kasmira* (NGR)

#### Bothriocephalidea

Minimized number of taxa: 1

Number of SLIPs: 0

Number of non-SLIP taxa: 0

Undistinguishable larval taxa: 1

unidentified family: unidentified species, larvae

Caesio cuning

Lutjanus adetii

Lutjanus kasmira

Nemipterus furcosus

#### Tetraphyllidea

Minimized number of taxa: 1

Number of SLIPs: 0

Number of non-SLIP taxa: 0

Undistinguishable larval taxa: 1

unidentified family: unidentified species, larvae

Caesio cuning

Etelis carbunculus

Lutjanus argentimaculatus

Lutjanus kasmira

Lutjanus quinquelineatus

Lutjanus vitta

Nemipterus furcosus

Pentapodus aureofasciatus

Pentapodus nagasakiensis *

Pristipomoides argyrogrammicus

Pristipomoides auricilla *

#### Trypanorhyncha

Minimized number of taxa: 8

Number of SLIPs: 7

Number of SLIPs: total 7; Lutjanidae: reef: 2-2*, deep-sea: 0-0*, all 2-2*; Nemipteridae: 6-6*.

Number of non-SLIP taxa: 0

Undistinguishable larval taxa: 1

Note: for minimizing number of taxa, we considered that all unproductive cysts from deep-sea lutjanids corresponded to 1 taxon and was distinct from the four other cyst-producing species.

**Laci:***Callitetrarhynchus gracilis*, larvae

Lutjanus vitta

*Nemipterus furcosus* (NHR)

**Laci:***Floriceps minacanthus*, larvae

Nemipterus furcosus

**Laci:***Pseudolacistorhynchus heroniensis*, larvae

*Lutjanus vitta* (NHR)

**Otob:***Otobothrium mugilis*, larvae

*Nemipterus furcosus* (NHR)(NGR)

**Tent:***Nybelinia goreensis*, larvae

Nemipterus furcosus

**Tentaculariidae:***Nybelinia indica*, larvae

*Nemipterus furcosus* (NHR)(NGR)

**Tentaculariidae:***Nybelinia queenslandensis*, larvae

*Nemipterus furcosus* (NHR)(NGR)

unidentified family: unidentified species, larvae

Etelis carbunculus

Etelis coruscans

Pristipomoides argyrogrammicus

Pristipomoides auricilla *

#### Nematoda

Minimized number of taxa: 17

Number of SLIPs: total 9; Lutjanidae: reef: 2-2*, deep-sea: 4-4*, all 6-6*; Nemipteridae: 3-3*.

Number of non-SLIP taxa: 6

Undistinguishable larval taxa: 1 (anisakids, gnathostomatids)

Note: for minimizing number of taxa, we considered that unidentified anisakids corresponded to one of the 3 identified larval anisakid genera; *Camallanus* sp. and unidentified camallanids were counted as a single species; *Huffmanela* sp. was distinguished as different; the *Raphidascaris (Ichthyascaris)* sp. from reef lutjanids was different from that from deep-sea.

**Anis:***Anisakis* sp., larvae

Nemipterus furcosus

**Anis:***Hysterothylacium* sp., larvae

Nemipterus furcosus

**Anis:***Raphidascaris (Ichthyascaris) etelidis*

Etelis coruscans

Pristipomoides filamentosus

**Anis:***Raphidascaris (Ichthyascaris) nemipteri*

Nemipterus furcosus

**Anis:***Raphidascaris (Ichthyascaris)* sp.

Etelis carbunculus

Lutjanus vitta

**Anis:***Terranova* sp., larvae

Lutjanus vitta

**Anis:** unidentified species, larvae

Lutjanus adetii

Lutjanus fulviflamma

Lutjanus kasmira

Lutjanus quinquelineatus

Lutjanus rivulatus *

Pentapodus aureofasciatus

Pentapodus nagasakiensis *

Pristipomoides argyrogrammicus

Pristipomoides auricilla *

Scolopsis taenioptera *

**Cama:***Camallanus carangis*

Nemipterus furcosus

**Cama:***Camallanus* sp.

Pristipomoides filamentosus

**Cama:** unidentified species

Lutjanus vitta

Pristipomoides auricilla *

Scolopsis bilineata

**Capi:***Pseudocapillaria novaecaledoniensis*

Pristipomoides argyrogrammicus

**Cucu:***Cucullanus bourdini*

Pristipomoides auricilla *

Pristipomoides filamentosus

**Cucu:***Dichelyne etelidis*

Etelis carbunculus

Etelis coruscans

**Gnto:** unidentified species

Nemipterus furcosus

**Phil:***Philometra brevicollis*

Lutjanus vitta

**Phil:***Philometra mira*

Lutjanus vitta

**Phys:** unidentified species

Nemipterus furcosus

**Tric:***Huffmanela branchialis*

Nemipterus furcosus

**Tric:***Huffmanela* sp.

Pentapodus aureofasciatus

#### Hirudinea

Minimized number of taxa: 1

Number of SLIPs: 0

Number of non-SLIP taxa: 1

Undistinguishable larval taxa: 0

**Pisc:***Trachelobdella* sp., juvenile

Lutjanus rivulatus *

Scolopsis bilineata

#### Turbellaria

Minimized number of taxa: 1

Number of SLIPs: 0

Number of non-SLIP taxa: 1

Undistinguishable larval taxa: 0

unidentified family: unidentified species

Nemipterus furcosus

#### Acanthocephala

Minimized number of taxa: 1

Number of SLIPs: 0

Number of non-SLIP taxa: 1

Undistinguishable larval taxa: 0

unidentified family: unidentified species

Etelis carbunculus

### Appendix 3. Material deposited

#### Pisces

*Nemipterus furcosus*, MNHN 2005–0768, 2006–1330.*Pentapodus aureofasciatus*, MNHN 2004–1168, 2004–1169, 2004–2164, 2004–2172.

#### Isopoda

**Aegi:***Aega musorstom* ex *Pr. filamentosus*, MNHN Is6295, Is6296, Is6297, Is6298.

**Cora:***Argathona macronema* ex *Lu. monostigma*, MNHN Is6299.

**Cymo:***Anilocra gigantea* ex *Et. carbunculus*, MNHN-IU-2009-1710, IU-2009-1712; ex *Et. coruscans*, MNHN Is6293, MNHN-IU-2009-1711; ex *Pr. filamentosus*, MNHN Is6292.

**Cymo:***Anilocra longicauda* ex *Pr. argyrogrammicus*, MNHN Is6294.

#### Copepoda

**Cali:***Caligus brevis*, ex *Et. coruscans*, MNHN Cp8059, BMNH 2010.767–769; ex *Et. carbunculus*, BMNH 2010.770–771.

**Diss:***Dissonus excavatus*, ex *Ma. niger*, MNHN Cp2436, BMNH 2007.316–325.

**Hats:***Hatschekia clava*, ex *Lu. gibbus*, MNHN Cp8067.

**Hats:***Hatschekia tanysoma*, ex *Lu. fulviflamma*, MNHN Cp8068–8069.

**Penn:***Lernaeolophus sultanus*, ex *Pr. filamentosus*, BMNH 2010.750.

**Lerp:***Parabrachiella lutiani*, ex *Lu. argentimaculatus*, MNHN Cp8060, BMNH 2010.786–791.

#### Monogenea

**Ancy:***Haliotrematoides lanx* ex *Lu. quinquelineatus*, slides MNHN JNC1590, JNC2145, JNC2140, USNPC 101344–5, BMNH 2008.11.19.36–37.

**Ancy:***Haliotrematoides longitubocirrus* ex *Lu. vitta*, slides MNHN JNC2306, USNPC 101349, 101350–352, BMNH 2008.11.19.38–39; ex *Lu. russellii*, MNHN JNC1584, USNPC 101347; ex. *Lu. fulvus*, USNPC 101346; ex. *Lu. quinquelineatus*, MNHN JNC1588, USNPC 101348.

**Ancy:***Haliotrematoides novaecaledoniae* ex *Lu. argentimaculatus*, slides MNHN JNC2332, USNPC 101337, BMNH 2008.11.19.24–27.

**Ancy:***Haliotrematoides patellacirrus* ex *Lu. russellii*, slides MNHN JNC1582, JNC1583, JNC1584, JNC1585, USNPC 101338, BMNH 2008.11.19.28–29; ex *Lu. fulviflamma*, slide MNHN JNC2531; ex *Lu. fulvus*, slides MNHN JNC1591, JNC1592, USNPC 101341, BMNH 2008.11.19.32–33; ex *Lu. quinquelineatus*, slides MNHN JNC2146, JNC2147, JNC2142, JNC2141, JNC2144, USNPC 101342, 101343, BMNH 2008.11.19.34–35; ex. *Lu. vitta*, MNHN 2470.

**Ancy:***Haliotrematoides potens* ex *Lu. argentimaculatus*, slides MNHN JNC2332, USNPC 101336, BMNH 2008.11.19.23.

**Ancy:***Haliotrematoides tainophallus* ex *Lu. fulviflamma*, slides MNHN JNC2531.

**Caps:***Benedenia elongata* ex gills *Et. carbunculus* (fish JNC2459) SAMA AHC 35066 (6 slides).

**Caps:***Benedenia elongata* ex gills *Et. coruscans* (fish JNC2448) MNHN JNC2448 A1 (1 slide).

**Caps:***Benedenia elongata* ex gills *Pr. argyrogrammicus* (from fish JNC2449) MNHN JNC2449 B1 (1 slide), (from fish JNC2603) MNHN JNC2603 A3 (1 slide), SAMA AHC 35067 (2 slides), (from fish JNC2604) MNHN JNC2604 A1 (1 slide), (from fish JNC2729) AHC 35068 (1 slide of a single specimen that was ‘slivered’; sliver fixed in 95% ethanol; possibly conspecific with Capsalidae sp. 17 of Perkins 2010), (from fish JNC2730) SAMA AHC 35069 (1 slide).

**Caps:** Capsalidae sp. 7 (see Perkins 2010) ex branchiostegal membranes *Lu. vitta* (from fish JNC2686) SAMA AHC 29706 (1 slide).

**Caps:***Lagenivaginopseudobenedenia etelis* ex gills *Et. coruscans* (from fish JNC2616) MNHN JNC2616 A1 (1 slide), JN111, JN115, JN119.

**Caps:***Metabenedeniella* sp. (see Perkins 2010) ex pectoral fins *Lu. argentimaculatus* (from fish JNC2735) SAMA AHC 29714 (3 slides).

**Caps:***Pseudonitzschia uku* (see Perkins et al. 2009) ex gills *Aprion virescens* (from fish JNC1557) MNHN JNC1557 A1 (1 slide), SAMA AHC 35070 (2 slides).

**Caps:***Trilobiodiscus lutiani* (see Perkins 2010) ex gills *Lu. argentimaculatus* (from JNC2332), USNPC 101526 (4 slides), (from JNC2735) SAMA AHC 29713 (1 slide).

**Caps:** Capsalidae sp. 13 (see Perkins 2010) ex branchiostegal membranes *Ne. furcosus* (from fish JNC2692) SAMA AHC 29707 (2 slides), AHC 35073 (4 slides).

**Caps:** Capsalidae sp. 13 of Perkins (2010) ex gills *Ne. furcosus* (from fish JNC971) MNHN JNC971A6 (1 slide), SAMA AHC 35071 (2 slides), (from fish JNC2692) SAMA AHC 35072 (5 slides), (from fish JNC2693) SAMA AHC 35075 (4 slides), (from fish JNC2694) SAMA AHC 35076 (6 slides), (from fish JNC2695) SAMA AHC 35077 (2 slides).

**Caps:** Capsalidae sp. 13 of Perkins (2010) ex pelvic and anal fins *Ne. furcosus* (from fish JNC2694), SAMA AHC 35074 (3 slides).

**Dipl:***Diplectanum* cf. *curvivagina* ex *Pr. argyrogrammicus*, slides MHNH JNC2426, JNC2449, JNC2456, JNC2729, JNC2996.

**Dipl:***Diplectanum* cf. *opakapaka* ex *Pr. auricilla*, slides MHNH JNC2457.

**Dipl:***Diplectanum* sp. ex *Pr. filamentosus*, slides MHNH JNC2452, JNC2460.

#### Polyopisthocotylea

**Micr:***Allomicrocotyla* sp. ex. *Et. coruscans*, slides JN114.

Unidentified polyopisthocotylean ex *Lu. russellii*, slide JNC1582.

#### Digenea

**Acan:***Pleorchis uku* ex *Aprion virescens*, MNHN JNC2568.

**Acan:***Stephanostomum uku* ex *Ap. virescens*, MNHN JNC1557C.

**Cryp:***Adlardia novaecaledoniae* ex *Ne. furcosus*, MNHN JNC2291–1, 16, MNHN JNC2289-1–5, MNHN JNC2291-2–4, MNHN JNC2331B-1–4, MNHN JNC2288-1–3, MNHN JNC2398-1, 10; BMNH 2008.12.30.1–3.

**Cryp:***Euryakaina manilensis* ex *Lu. vitta*, MNHN JNC2285, MNHN JNC2286, MNHN JNC2306, MNHN JNC2470, MNHN JNC2686, MNHN JNC2862, MNHN JNC2887, MNHN JNC2897, MNHN JNC2898, MNHN JNC2900, MNHN JNC2902; BMHN 2010.4.23.1–12.

**Cryp:***Euryakaina marina* ex *Lu. fulviflamma*, MNHN JNC1269B.

**Cryp:***Retrovarium manteri* ex *Lu. argentimaculatus*, MNHN JNC 2735–2, BMNH 2012.2.15.7

**Cryp:***Retrovarium saccatum* ex *Lu. argentimaculatus*, MNHN JNC 2735–3

**Cryp:***Siphoderina hirastricta* ex *Lu. argentimaculatus*, MNHN JNC 2735–1

**Cryp:***Varialvus charadrus* ex *Lu. quinquelineatus*, MNHN JNC2143; ex *Lu. vitta* MNHN JNC2285, MNHN JNC2688, MNHN JNC2689.

**Fell:***Tergestia magna* ex *Et. coruscans*, MNHN JNC 2616B-1, JNC2617B-1; ex *Pr. argyrogrammicus*, MNHN JNC2820B-1.

**Hemi:***Ectenurus trachuri* ex *Ne. furcosus*, MNHN JNC2586-1.

**Lepo:***Lepidapedoides kalikali* ex *Pr. auricilla*, MNHN JNC2457-1, JNC2468-1, BMNH 2012.2.15.6.

**Monr:***Allobacciger macrorchis* ex *Sc. bilineata*, MNHN JNC2522-1, BMNH 2012.2.15.16.

**Opec:***Hamacreadium mutabile* ex *Lu. adetii*, MNHN JNC3021-1; ex *Lu. argentimaculatus*, MNHN JNC2735-2, BMNH 2012.2.15.15; ex *Lu. fulviflamma*, MNHN JNC2531-1, BMNH 2012.2.15.13; ex *Lu. kasmira*, MNHN JNC2708-1-2, BMNH 2012.2.15.14; ex *Lu. quinquelineatus*, MNHN JNC 2142–1; ex *Lu. russellii*, MNHN JNC2191-1; ex *Lu. vitta*, MNHN JNC 2285–1, MNHN 2306–1, MNHN JNC2470-1, MNHN JNC2686-1, MNHN JNC2900-1, MNHN JNC2896-1, MNHN JNC2898-1, MNHN JNC2899-1, BMNH 2012.2.15.9–12.

**Opec:***Macvicaria jagannathi* ex *Ne. furcosus*, MNHN JNC2331A-1–3, JNC2366A-1., BMNH 2009.2.12.11-14.

**Opec:***Neolebouria blatta* ex *Et. carbunculus*, MNHN JNC2427; ex *Pr. argyrogrammicus,* MNHN JNC2464-1, MNHN JNC2426, MNHN JNC2456, MNHN JNC2461, MNHN JNC2464-66, MNHN JNC2604-05, MNHN JNC 2729, MNHN JNC2995-1, MNHN JNC2996A-1; BMNH 2009.4.7.1–7, 2012.2.15.8.

**Opec:***Neolebouria lineatus* ex *Ne. furcosus*, MNHN JNC2398-1–2; BMNH 2009.2.12.15.

**Scle:***Prosogonotrema bilabiatum* ex *Lu. adetii*, MNHN JNC3022-1.

**Tran:***Transversotrema borboleta* ex *Lu. kasmira*, MNHN JNC2708.

#### Trypanorhyncha

**Laci:***Callitetrarhynchus gracilis* ex *Ne. furcosus*, slide MNHN JNC2596.

**Laci:***Floriceps minacanthus* ex *Ne. furcosus*, slide MNHN JNC3019.

**Otob:***Otobothrium mugilis* ex *Ne. furcosus*, slides MNHN JNC2610, JNC2586.

**Tent:***Nybelinia goreensis* ex *Ne. furcosus*, slide MNHN JNC2612.

**Tent:***Nybelinia indica* ex *Ne. furcosus*, slides MNHN JNC2288, JNC2611, JNC3016.

#### Nematoda

**Anis:***Raphidascaris (Ichthyascaris) etelidis* ex *Et. coruscans*, MNHN JNC2616, JNC2617, JNC2623, HCIP N-969; ex *Pr. filamentosus*, MNHN JNC2460.

**Anis:***Raphidascaris (Ichthyascaris) nemipteri* ex *Ne. furcosus*, MNHN JNC218, JNC330, JNC214, JNC217, JNC278, JNC311, HCIP N-836.

**Cama:***Camallanus carangis* ex *Ne. furcosus*, MNHN JNC276, JNC280, JNC465, JNC467, JNC1261, HCIP N-859.

**Capi:***Pseudocapillaria novaecaledoniensis* ex *Pr. argyrogrammicus*, MNHN JNC2604, HCIP N-930.

**Cucu:***Cucullanus bourdini* ex *Pr. auricilla* MNHN JNC2457, ex *Pr. filamentosus* JNC2460, HCIP N-949.

**Cucu:***Dichelyne etelidis* ex *Et. carbunculus*, MNHN JNC2459, HCIP N-948; ex *Et. coruscans*, MNHN JNC2617.

**Phil:***Philometra brevicollis* ex *Lu. vitta*, MNHN JNC2901, JNC2038, HCIP N-967.

**Phil:***Philometra mira* ex *Lu. vitta*, MNHN JNC2901, HCIP N-968.

**Tric:***Huffmanela branchialis* ex *Ne. furcosus*, NHN JNC216, HCIP N-816, BMNH 2004.2.18.1, SAMA AHC 32856.

**Tric:***Huffmanela* sp. ex *Pe. aureofasciatus*, MNHN JNC1040.

### Abbreviations

NGR: New geographic record; NHR: New host record; HPC: Host-parasite combination; SLIP: Species-level identified parasite; SLIP-HPC: Species-level identified parasite – host-parasite combination.

### Institutional abbreviations

BMNH: Natural History Museum, London, United Kingdom; HCIP: Helminthological Collection, Institute of Parasitology, Biology Centre, Academy of Sciences of the Czech Republic, České Budějovice, Czech Republic; MNHN: Muséum National d’Histoire Naturelle, Paris, France; SAMA AHC: South Australian Museum Adelaide, Australian Helminthological Collection, Adelaide, Australia; USNPC: United States National Parasite Collection, Beltsville, USA.

### Abbreviations for higher parasite taxa

The following abbreviations are used in Tables and Appendices.: ; For all: ; Unid: Unidentified family; Isop: Isopoda; Families: ; Aegi: Aegidae; Cora: Corallanidae; Cymo: Cymothoidae; Gnat: Gnathiidae; Cope: Copepoda; Families: ; Cali: Caligidae; Diss: Dissonidae; Hats: Hatschekiidae; Lerp: Lernaeopodidae; Penn: Pennellidae; Mono: Monogenea; Monop: Monopisthocotylea; Poly: Polyopisthocotylea; Families: ; Ancy: Ancyrocephalidae; Caps: Capsalidae; Dipl: Diplectanidae; Gyro: Gyrodactylidae; Micr: Microcotylidae; Dige: Digenea; Families: ; Acan: Acanthocolpidae; Cryp: Cryptogonimidae; Didy: Didymozoidae; Fell: Fellodistomatidae; Hemi: Hemiuridae; Leci: Lecithasteridae; Lepo: Lepocreadiidae; Monr: Monorchiidae; Opec: Opecoelidae; Scler: Sclerodistomidae; Tran: Transversotrematidae; Tryp: Cestoda Trypanorhyncha; Families: ; Laci: Lacistorhynchidae; Otob: Otobothriidae; Tent: Tentaculariidae; Both: Cestoda Bothriocephalidea (no family identified); Tetr: Cestoda Tetraphyllidea (no family identified); Nema: Nematoda; Families: ; Anis: Anisakidae; Cama: Camallanidae; Capi: Capillariidae; Cucu: Cucullanidae; Gnto: Gnathostomatidae; Phil: Philometridae; Phys: Physalopteridae; Tric: Trichosomoididae; Hiru: Hirudinea (no family identified); Family: ; Pisc: Piscicolidae; Turb: ‘Turbellaria’; Acantho: Acanthocephala (no family identified).

### Competing interests

The authors declare that they have no competing interests.

### Authors’ contributions

JLJ collected fish and parasites and compiled and compared results. JLJ IB GAB RAB TLM FM JPT IDW identified parasites. GAB JLJ made figures. JLJ IB GAB RAB TLM FM JPT IDW wrote the paper. Authors are in alphabetical order, except JLJ. All authors read and approved the final manuscript.

### Authors’ information

JLJ: specialist of phylogeny and taxonomy of parasitic worms, mainly monogeneans; professor and curator of the collections of parasitic worms of MNHN, Paris, France, has spent more than eight years (2003–2011) in Nouméa, New Caledonia, South Pacific, collecting and studying parasites from fish; UMR 7138 Systématique, Adaptation, Évolution, Muséum National d’Histoire Naturelle, Case postale 51, 55, rue Buffon, 75231 Paris cedex 05, France.

IB: specialist of phylogeny and taxonomy of parasitic worms, including trypanorhynch cestodes; Department of Veterinary Science, University of Melbourne, Veterinary Clinical Centre, Werribee 3030, Victoria, Australia.

GAB: specialist of all aspects of biology and taxonomy of copepods, including parasitic groups; Department of Zoology, Natural History Museum, Cromwell Road, London SW7 5BD, UK.

RAB: specialist of phylogeny and taxonomy of parasitic worms, mainly digeneans and cestodes; Department of Zoology, Natural History Museum, Cromwell Road, London SW7 5BD, UK.

TLM: specialist of phylogeny and taxonomy of digeneans, especially cryptogonimids; Biodiversity Program, Queensland Museum, PO Box 3300, South Brisbane, Queensland, 4101 Australia

FM: specialist of biology and taxonomy of parasitic nematodes; Institute of Parasitology, Biology Centre, Academy of Sciences of the Czech Republic, Branišovská 31, 370 05 České Budějovice, Czech Republic.

JPT: specialist of taxonomy and biogeography of parasitic isopods; Équipe Adaptation écophysiologique et Ontogenèse, UMR 5119 (CNRS-UM2-IRD-UM1-IFREMER), Université Montpellier 2, Place Eugène Bataillon, 34095 Montpellier cedex 05, France.

IDW: specialist of biology, taxonomy and phylogeny of monogeneans, including capsalids; Monogenean Research Laboratory, The South Australian Museum, Adelaide 5000, & Marine Parasitology Laboratory, & Australian Centre for Evolutionary Biology and Biodiversity, The University of Adelaide, North Terrace, Adelaide 5005, South Australia, Australia.
